# Adopting electric school buses in the United States: Health and climate benefits

**DOI:** 10.1073/pnas.2320338121

**Published:** 2024-05-20

**Authors:** Ernani F. Choma, Lisa A. Robinson, Kari C. Nadeau

**Affiliations:** ^a^Department of Environmental Health, Harvard T.H. Chan School of Public Health, Boston, MA 02115; ^b^Center for Health Decision Science, Harvard T.H. Chan School of Public Health, Boston, MA 02115

**Keywords:** transportation, air pollution, benefit–cost analysis, climate change, risk assessment

## Abstract

Electric school buses have been proposed as an alternative to reduce the health and climate impacts of the current U.S. school bus fleet, of which a substantial share are highly polluting old diesel vehicles. However, the climate and health benefits of electric school buses are not well known. As they are substantially more costly than diesel buses, assessing their benefits is needed to inform policy decisions. We assess the health benefits of electric school buses in the United States from reduced adult mortality and childhood asthma onset risks due to exposure to ambient fine particulate matter (PM_2.5_). We also evaluate climate benefits from reduced greenhouse-gas emissions. We find that replacing the average diesel bus in the U.S. fleet in 2017 with an electric bus yields $84,200 in total benefits. Climate benefits amount to $40,400/bus, whereas health benefits amount to $43,800/bus due to 4.42*10^−3^ fewer PM_2.5_-attributable deaths ($40,000 of total) and 7.42*10^−3^ fewer PM_2.5_-attributable new childhood asthma cases ($3,700 of total). However, health benefits of electric buses vary substantially by driving location and model year (MY) of the diesel buses they replace. Replacing old, MY 2005 diesel buses in large cities yields $207,200/bus in health benefits and is likely cost-beneficial, although other policies that accelerate fleet turnover in these areas deserve consideration. Electric school buses driven in rural areas achieve small health benefits from reduced exposure to ambient PM_2.5_. Further research assessing benefits of reduced exposure to in-cabin air pollution among children riding buses would be valuable to inform policy decisions.

## Significance

Whether to replace diesel school buses with cleaner electric models can be a difficult decision for local, state, and federal officials. Electric buses are expensive and their health benefits are not well known. We estimate the benefits of replacement across geographic areas to inform these decisions. When electric buses replace old diesel vehicles in large cities, health benefits associated with reduced mortality and childhood asthma total $207,200/bus. Climate benefits amount to $40,400/bus. These benefits likely exceed replacement costs. Because old buses are a large share of the current fleet, replacing them can substantially improve social welfare. Such improvements may be particularly important in low-income environmental justice communities, where budgets are often tight and health status is a major concern.

The transportation sector is the largest contributor to U.S. greenhouse-gas (GHG) emissions (1). Vehicle emissions are also an important contributor to ambient air pollution, causing substantial health effects (2–5). Several recent studies have attributed roughly 20,000 deaths per year in the United States to vehicle emissions, despite recent decreases (2–5). Around 90% of this mortality burden is due to exposure to ambient fine particulate matter (PM_2.5_), with ozone representing the remaining 10% (3, 6, 7). Ambient PM_2.5_ is the environmental exposure responsible for the largest mortality burden in the United States (8, 9). PM_2.5_ is emitted directly from vehicles (primary PM_2.5_) or is formed in the atmosphere after vehicle emissions of precursor gases such as nitrogen oxides (NO_x_), sulfur dioxide (SO_2_), ammonia (NH_3_), and volatile organic compounds (VOCs).

About half a million school buses are in circulation in the United States (10), serving about 24 million students (11). While school buses are only one component of the transportation system, a substantial portion of the school bus fleet is composed of highly polluting old diesel vehicles (10, 12–14). Increased regulation of heavy-duty vehicles starting with model year (MY) 2007 led to large reductions in school bus emissions per mile between MYs 2005 and 2010 (12, 15), but the U.S. Environmental Protection Agency (EPA) estimates that about 40% of the school bus fleet is older than 11 y (10), suggesting that about 200,000 pre-2010 MY school buses were still in circulation in 2021. MY 2005 diesel school buses are estimated to emit, per mile driven, roughly 12 times more primary PM_2.5_, 4 times more NO_x_, and 5 times more VOCs than MY 2010 diesel school buses (12). Older buses are even more polluting. Primary PM_2.5_ emission factors per mile dropped by 100 times between MY 1990 and MY 2015 diesel school buses (12).

Electric vehicles (EVs) have received much attention in recent years as a crucial alternative to reduce climate and health impacts of transportation in the United States and globally (16–19). Although light-duty vehicles still account for the vast majority of EVs (16), the number of electric school buses in the United States has increased in recent years (20, 21). In June 2023, according to one estimate, a total of 2,277 electric school buses had been ordered, delivered, or were operating in the United States, roughly twice as many as a year earlier, but still accounting for only 0.5% of the school bus fleet (20).

The U.S. EPA’s new Clean School Bus Program will provide $5 billion over 5 y from 2022 to 2026 to replace current buses with cleaner and healthier zero- or low-emission models that reduce GHG emissions, ambient air pollution, and health risks to children (22). The program focuses on replacing diesel buses MY 2010 or older, but newer buses are eligible to be replaced by zero-emission vehicles in the case of fleets without pre-MY 2010 vehicles (23). EPA’s Clean School Bus Program prioritizes investments in low-income and disadvantaged communities, as part of the Federal government’s Justice40 initiative that aims to deliver 40% of the overall benefits to communities that are marginalized by underinvestment and overburdened by pollution (24–27). Air pollution levels are higher near highways (28) and transportation emissions are also important contributors to racial and ethnic disparities in PM_2.5_ exposure: Black and Hispanic Americans face roughly 40 and 30% higher exposure to PM_2.5_ from heavy-duty diesel than White Americans, and about twice as much exposure as their consumption causes (29). At a cost of $300,000 to $400,000 per electric bus (30, 31), $5 billion can replace only around 15,000 buses, which amounts to just 3% of the fleet and a small fraction of the roughly 200,000 highly polluting pre-2010 school buses in circulation. Replacing all pre-2010 diesel school buses in the United States would require a $60 billion to $80 billion investment, whereas electrifying the entire U.S. school bus fleet would cost roughly 2.5 times as much.

Electric buses save on fuel and maintenance costs, but a recent analysis including vehicle, fuel, maintenance, and insurance and fee costs suggests that, on average, the total cost of ownership is about $156,000 higher for electric buses in the absence of any subsidies, relative to purchasing a new diesel bus (30, 32). However, costs vary by school bus type and size, by the capacity of the battery required to drive the routes, the type of charging station and charging behavior, and fuel/electricity costs, among other factors (30, 32). Additionally, the number of electric bus models and the manufacturing capacity is expanding rapidly (33), which may reduce costs in the future. In addition to high upfront costs, a pilot project in three school districts in Massachusetts in 2016 to 2018 showed that early adopters faced many substantial challenges such as lack of reliability and difficulties with maintenance and technical assistance, and in managing charging (34). In particular, unmanaged charging led to much higher than expected electricity consumption and to an erosion of expected savings in operating costs; the project did not even attempt to implement a vehicle-to-grid/vehicle-to-building component due to the challenges and risks involved (34). With the recent implementation of EPA’s Clean School Bus Program, however, the electric sector pledged to work with school districts to support electrification (35).

An older analysis concluded that electric school buses were cost-beneficial, but this conclusion was largely due to annual vehicle-to-grid revenues exceeding $15,000 per year based on frequency regulation market prices for a recently established market for the PJM (a regional transmission organization) grid and assuming that electric buses would be performing vehicle-to-grid services close to 90% of the time (36). More recent estimates suggest roughly between $40 and $400 per EV in vehicle-to-grid benefits (37, 38), a small benefit compared to the average $156,000 cost differential. The authors also suggest potentially large variation in vehicle-to-grid benefits, making it difficult for school districts to rely on revenues, even if they were larger, on average, for electric school buses.

As electric buses are more costly, understanding their potential health and climate benefits is crucial to inform policy decisions regarding their adoption. Research has focused on EVs’ potential to reduce transportation’s climate impacts (17), but urban light-duty EVs in the United States also lead to large public health benefits (39). This is largely because emissions in urban areas—such as tailpipe emissions of urban vehicles—affect a large population, causing large health impacts (2, 4, 39). Although less research has been conducted for electric heavy-duty vehicles, recent studies also suggest their use in U.S. urban areas has large health benefits (40, 41) and a study for Chicago showed that the largest benefits accrued to Black and Hispanic/Latino Americans, helping reduce racial and ethnic air pollution disparities (40). Nevertheless, previous studies did not assess school buses, and their health benefits are not known. Holland et al. analyzed urban transit buses, but their results are not applicable to school buses due to differences in vehicle types and emissions; furthermore, they relied on relatively old emission factors for vehicles (pre-2013) and power plants (2017) (41).

Our main objective is to assess the health and climate benefits of electric school bus adoption in the United States. We estimate per-mile health benefits of replacing diesel school buses with electric school buses in each of the 3,108 counties in the contiguous United States, covering diesel school bus emissions of primary PM_2.5_, NO_x_, NH_3_, SO_2_, and VOCs, and power plant emissions of SO_2_ and NO_x_. We also map our county-level results to school districts to provide school district-level estimates. We supplement these estimates with climate benefits from reductions in GHG emissions. We estimate benefits of electric buses replacing four types of diesel buses: the fleet average in 2017 for each county, and diesel buses of MYs 2005, 2010, and 2020. We account for health benefits from reduced chronic exposure to ambient PM_2.5_, including its effects on mortality among adults and on new asthma cases among children aged 0 to 17 y. We focus on chronic exposure to PM_2.5_ since its impacts are much larger than those of acute exposure; about 10 times as large even in urban areas in China, where haze episodes can be severe (42). We report per-mile results, reflecting the value per each mile driven at present time, as well as per-vehicle results, which assume school buses are driven a total of 190,134 miles over the next 13.5 y (30) and that emissions are uniformly distributed along the 13.5-y period, calculating the present value of impacts that occur in the future.

We aim to provide an estimate of the health and climate benefits of electric school buses to better inform local, state, and federal policymakers as they make difficult decisions when allocating scarce resources. In the discussion section, we also compare these benefits with costs. Our work focuses exclusively on school buses, and we refer to them simply as “buses” for the remainder of the manuscript.

## Results

**Fig. 1.**
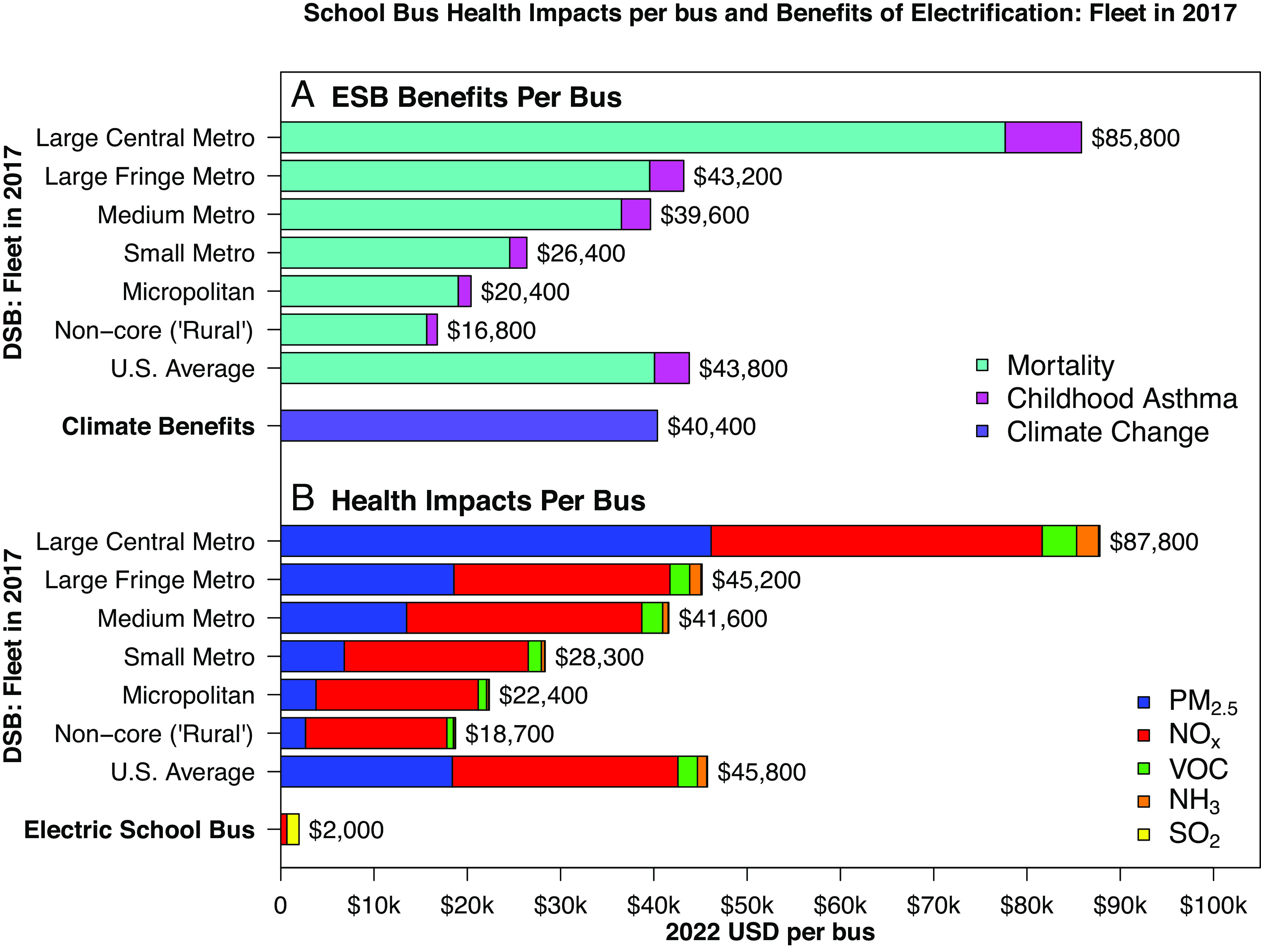
(*A*) Health benefits of school bus electrification, when replacing the average diesel bus in the fleet in 2017, by driving location and outcome. (*B*) Health impacts of the school bus fleet in 2017, by driving location and pollutant species. Locations are classified using NCHS’s Urban–Rural classifications (43). DSB: Diesel School Bus. ESB: Electric School Bus.

We find that the benefit of replacing the average diesel bus in U.S. fleet in 2017 with an electric bus is $84,200 per bus, with $43,800 in health benefits and $40,400 in climate benefits due to a reduction in GHG emissions of 181 t of CO_2_-eq. per bus (Fig. 1). The $43,800 in health benefits are due to a reduction of 4.42*10^−3^ in PM_2.5_-attributable deaths (with an economic value of $40,000, or 91% of the total) and a reduction of 7.42*10^−3^ in PM_2.5_-attributable new childhood asthma cases (with an economic value of $3,700, or 9% of the total) (*SI Appendix*, Figs. S1–S4).

Health impacts of electric buses are very small in comparison to those of diesel buses. We estimate that the entire school bus fleet in 2017 was responsible for 170 PM_2.5_-attributable deaths and 280 PM_2.5_-attributable new childhood asthma cases per year; however, if the entire school bus fleet were electrified, it would be responsible for just 7 PM_2.5_-attributable deaths and 12 PM_2.5_-attributable new childhood asthma cases per year. School buses were driven for 6.8 billion miles in 2017 (44), and we attribute 2.43 deaths and 4.08 new asthma cases per 100 million miles driven by the average diesel school bus in the U.S. fleet in 2017; replacing these buses with electric buses would drop these numbers to 0.10 and 0.17, respectively (*SI Appendix*, Figs. S3–S4).

**Fig. 2.**
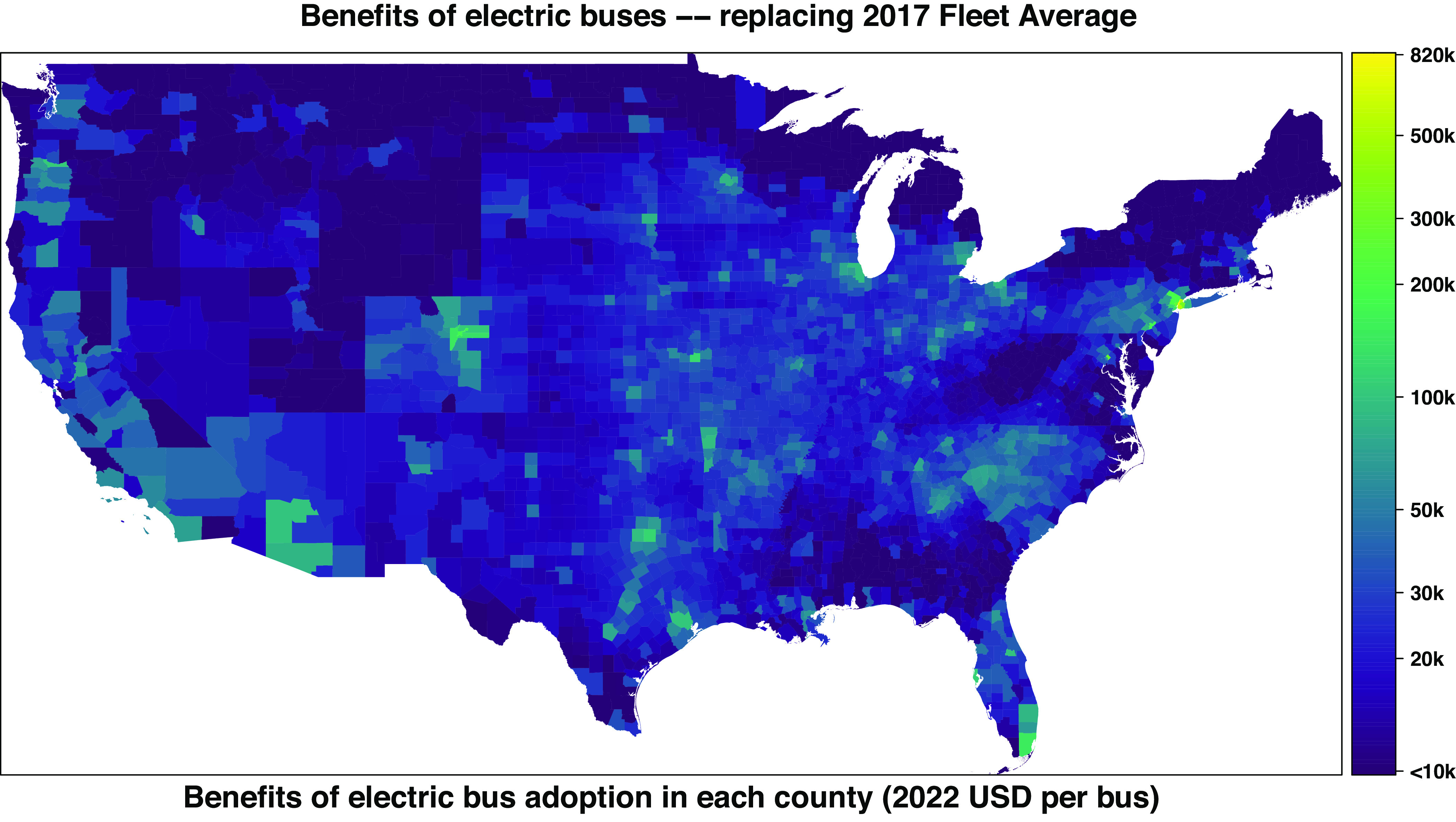
Health benefits of electric school bus adoption in each county, if they replace the average diesel school bus in each county’s fleet in 2017. Values smaller than $10,000 are not shown (n = 448 counties), including three counties with negative benefits varying between -$300 and -$800. County geographical boundaries are from the U.S. Census Bureau (47).

However, the health benefits of electric buses vary substantially depending on which diesel buses are being replaced, with diesel bus MY and location of driving being two key determinants of benefits. Health benefits are larger if diesel buses in more densely populated areas are replaced, since health impacts per mass emitted in these areas are much higher as they cause exposure in a larger number of people (*SI Appendix*, Figs. S5–S9). Health benefits of replacing the average bus in each county’s fleet in 2017 vary from $822,000 in New York County, NY to -$800 in Washington County, ME (Fig. 2). Mapping these results to school districts (45, 46) (*SI Appendix*, section 1.7), benefits vary between $503,300 for the New York City Department of Education to -$800 for various school districts located in Washington County, ME (Dataset S4). When we aggregate counties by degree of urbanization using the National Center for Health Statistics classification (43), health benefits of replacing the average bus in each area’s fleet in 2017 vary from $85,800 in large central metropolitan counties to $16,800 in noncore (“rural”) counties, which are the most rural counties, located outside metropolitan or micropolitan areas. Electrification of school buses in large metropolitan areas, which we define as those with population larger than 1 million, represented by central and large fringe metro counties, leads to health benefits of $62,200 per bus. However, some metropolitan areas still derive much larger health benefits than others, varying between $11,200 and $135,500 for electric buses driven in the Hartford and New York City metropolitan areas, respectively. According to National Emissions Inventory (NEI) 2017 data (44), 46% of the school bus vehicle miles traveled occurred in large metropolitan areas (20% in large central counties).

**Fig. 3.**
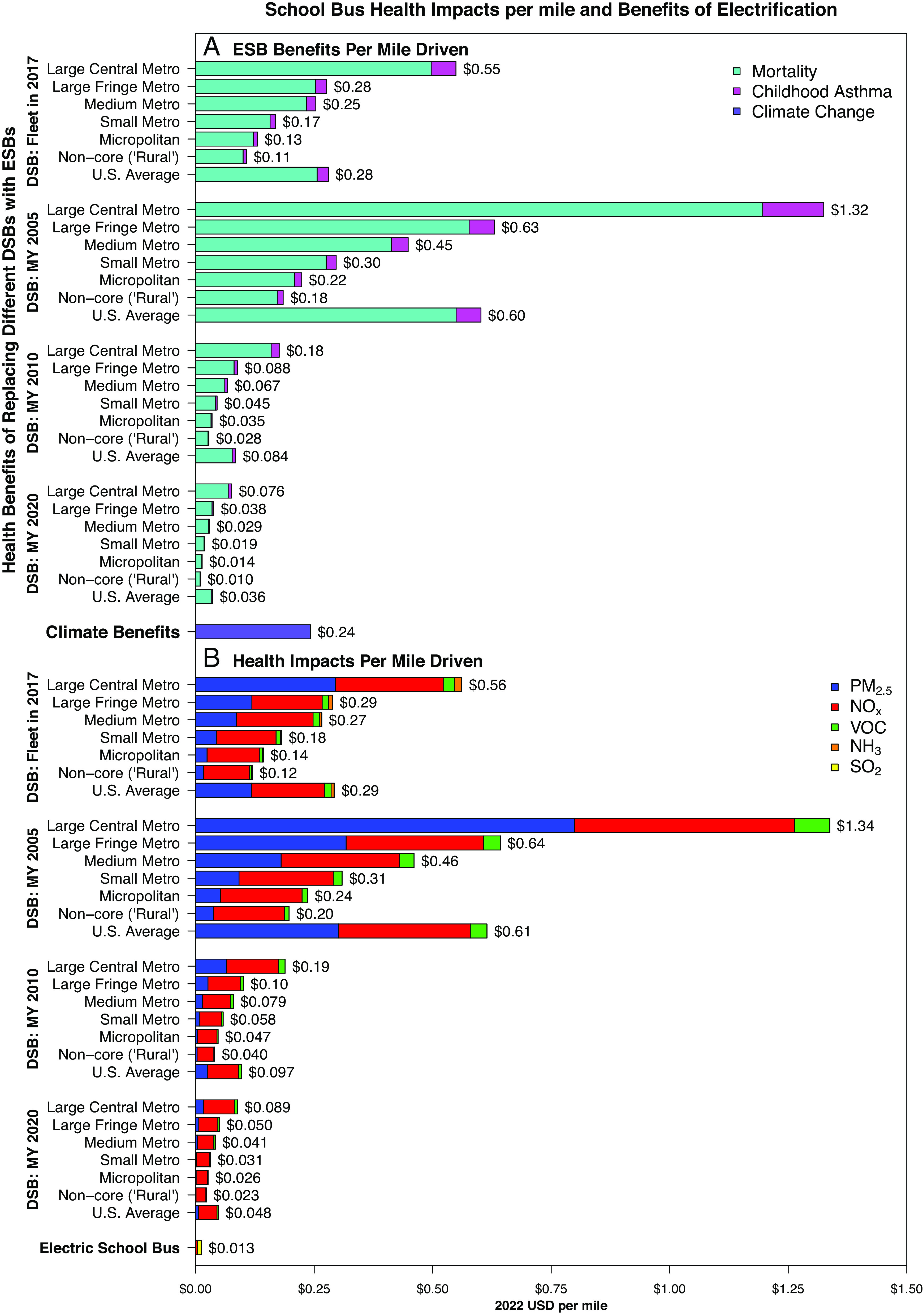
(*A*) Per-mile health benefits of school bus electrification, by bus MY, driving location, and outcome. (*B*) Per-mile health impacts of school buses, by bus MY, driving location, and pollutant species. Locations are classified using NCHS’s Urban–Rural classifications (43). DSB: Diesel School Bus. ESB: Electric School Bus.

Health benefits of electric buses are also much larger if older diesel buses are replaced, since they cause much higher health impacts (Fig. 3 and *SI Appendix*, Fig. S10). Benefits of replacing MY 2005 diesel buses with electric buses are roughly 7 times larger than replacing MY 2010 diesel buses, 16 to 18 times larger than replacing MY 2020 diesel buses, and 1.7 to 2.4 times as large as replacing the average school bus in the fleet in 2017. Each mile driven by a MY 2005 diesel bus in large central metropolitan areas that is replaced with an electric mile results in a health benefit of $1.32 (Fig. 3), because MY 2005 diesel buses cause $1.34 in health impacts per mile driven whereas electric buses cause just $0.013. We primarily report per-mile benefits since it is unlikely that a new electric bus would replace only miles driven by MY 2005 vehicles—as these vehicles are old and most likely near the end of their service lives—but the higher the share of old miles replaced by an electric bus, the larger the benefit achieved over its lifetime. An electric bus that entirely replaced MY 2005 diesel miles driven in central large metropolitan areas would achieve $207,200 in health benefits (*SI Appendix*, Fig. S10).

A substantial proportion of the large health impacts of old diesel buses driven in densely populated areas are due to primary PM_2.5_ emissions and accrue largely to the local population. The impact per mass of primary PM_2.5_ emitted is much larger in densely populated areas—exhibiting much larger spatial variability than the impacts per mass of NO_x_ emitted (*SI Appendix*, Figs. S5–S8)—and older diesel buses emit more primary PM_2.5_ per mile driven. Impacts of ground-level primary PM_2.5_ emissions also occur much closer to the source (2). As a consequence, 85% of the health impacts of MY 2005 diesel buses driven in large metropolitan areas accrue to the population living in the same metropolitan area, although this varies between 56% and 97% among the different metropolitan areas, in part due to variation in geographical size and population density. For MY 2005 diesel bus driven in central counties, 79% of impacts accrue to the population in the same county, a share that varies between 27% and 96% for various central areas.

**Fig. 4.**
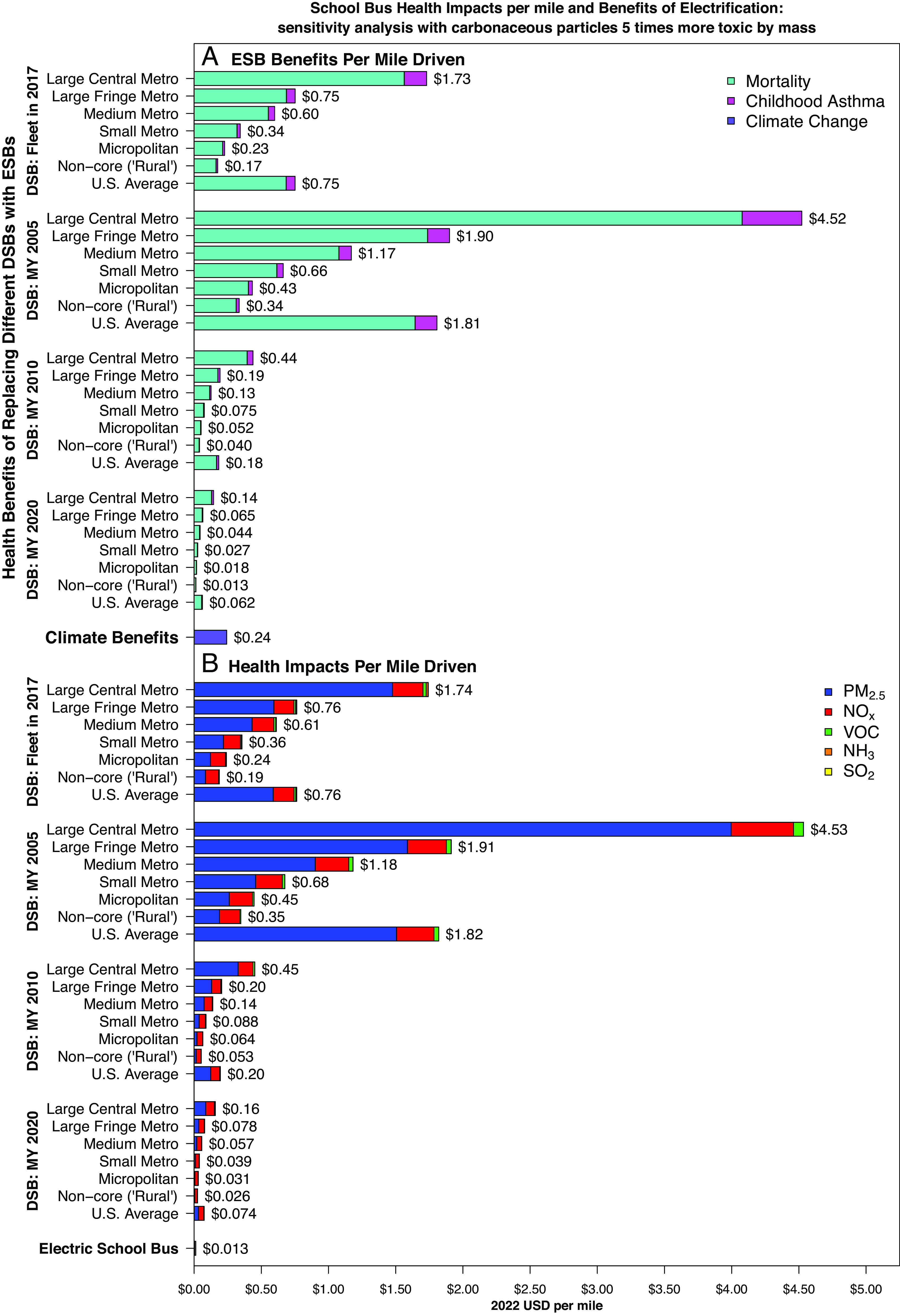
Sensitivity analysis assuming carbonaceous particles are five times more toxic than the ambient mix, by mass. (*A*) Per-mile health benefits of school bus electrification, by bus MY, driving location, and outcome. (*B*) Per-mile health impacts of school buses, by bus MY, driving location, and pollutant species. Locations are classified using NCHS’s Urban–Rural classifications (43). DSB: Diesel School Bus. ESB: Electric School Bus.

In a sensitivity analysis where we treat carbonaceous particles as five times more toxic than the ambient mix by mass (*Materials and Methods*), electric bus benefits increase by roughly a factor of 3 when older buses in metropolitan areas are replaced, and by roughly a factor of 1.5 to 2 when diesel buses in rural areas or newer buses are replaced (Fig. 4 and *SI Appendix*, Fig. S11). Benefits are always larger if primary PM_2.5_ from diesel buses is more toxic than average, but they increase more when urban and old diesel buses are replaced because primary PM_2.5_ is responsible for a larger share of their total health impacts.

## Discussion

Our results indicate that replacing older diesel school buses driven in large metropolitan areas leads to large health benefits—exceeding $200,000 per bus if electric buses replace miles driven by diesel buses of MY 2005 driven in central metropolitan areas—whereas replacing diesel buses of newer MYs or in areas with lower population density leads to much smaller health benefits. An electric school bus costs $156,000 more, on average, than a new diesel school bus in terms of lifetime costs, after accounting for fuel and maintenance savings of electric buses (32); supplementary materials *SI Appendix* suggest that this present value is in 2022 dollars (30), calculated using a 3% discount rate (48).

Therefore, electric school buses would be beneficial if health and climate benefits—which are externalities not accounted for in these costs—are larger than this cost differential of $156,000 per bus. As we estimate average climate benefits of $40,400 per electric bus, they would require about $115,000 in additional health benefits to be cost-beneficial, which is more than the overall average estimate per bus but less than the estimate for urban areas. Although we do not capture variability in climate benefits of electric buses due to, e.g., variation in diesel bus fuel economy and electricity grid emissions, it seems unlikely that climate benefits alone could counterbalance a large share of the $156,000 cost differential. In this case, it seems that health benefits are important to the decision of whether to adopt electric buses.

Our results capture only health benefits associated with reduced ambient PM_2.5_, but these benefits are large enough to suggest that replacing many old diesel buses driven in densely populated areas is cost-beneficial. However, due to potentially large variability in school bus costs [e.g., by bus school bus type and size, battery capacity and charging, fuel/electricity costs, among others (30, 32)] and health benefits, our results should be combined with additional data specific to each school district on cost factors and fleet characteristics to assess where school bus adoption is cost-beneficial and which areas derive the most benefits. A location-specific assessment of costs and benefits would also allow for a comparison between electric school bus adoption and other policies competing for resources in the transportation or other sectors covered by the local budget.

Electric buses achieve large benefits when they replace old diesel buses because old diesel buses are highly polluting. As emission factors for MY 2010 and newer diesel buses decreased very substantially, similarly large health benefits would be achieved by replacing old diesel buses with new diesel buses: Replacing MY 2005 with MY 2020 diesel buses achieves 90% of the health benefits of replacing the same MY 2005 diesel buses with electric buses. Therefore, policies that accelerate fleet turnover and remove old school buses from circulation, especially those driven in large metropolitan areas, also deserve consideration. Data from the U.S. EPA (10) suggest that about 40% of the fleet in 2021 was composed of pre-MY 2010 diesel buses, whereas a more recent sample of about 290,000 school buses with known MYs suggests about 25% of pre-MY 2010 in the fleet in 2022 (13). Even if old buses are used less frequently and drive fewer miles, they still likely account for a disproportionate amount of the total health impacts, since their impacts per mile are roughly 10 times larger.

About two-thirds of the U.S diesel school bus health impacts in 2017 were caused by those driven in large metropolitan areas. High population density in large metropolitan areas leads to many more people being exposed to diesel bus emissions. The intake fraction, defined as the fraction of the emissions that is inhaled by the population (49), can be orders of magnitude higher in large cities, relative to rural areas (39, 50). For old diesel buses in large metropolitan areas, the high impact per mass emitted is combined with larger emission factors; therefore, replacing a relatively small number of miles driven by them could lead to substantial public health benefits. While using federal funds to foster electric school bus adoption primarily in large metropolitan areas could raise concerns about the geographic distribution of these funds, our results suggest that differentiating policies by location may be appropriate since typically 80 to 85% of the benefits accrue to the local population. This is because primary PM_2.5_ accounts for a large share of impacts, especially of older vehicles, and the health impacts of ground-level primary PM_2.5_ emissions occur close to the source (2).

Electric school bus deployment leads to health benefits in almost all regions because they cause low health impacts ($0.013/mile), largely due to substantial decreases in grid emission factors achieved in the past decade and to further cuts projected throughout the electric bus lifetime (51). Holland et al. (41) estimated $0.11/mile (2014 USD) in climate and health impacts for electric transit buses using grid emissions for 2017. If we used their values for transit bus consumption (2.33 kWh/mile) and social cost of CO_2_ ($43.50 per metric ton, much less than current estimates), we would estimate $0.037/mile in climate and $0.019/mile in health impacts. These values are smaller than those estimated by Holland et al. mostly because we use projected grid emissions from 2023 to 2036, which are roughly 2, 3, and 4 times lower for GHGs, NO_x_, and SO_2_, respectively, relative to 2018 (51). Our previously estimated values of 2.2 attributable deaths per TWh in 2018 (39) are similar to Holland et al. (41) and to a recent estimate of 8,500 deaths attributable to power plant emissions in 2018 (3), considering that 4,200 TWh were generated that year (52). A limitation of our future estimates is that we do not capture changes in demographics, in the spatial distribution of grid emissions, or other factors that may affect the impacts per mass emitted in the future, but we assume these effects will be small compared to the large decreases in emission factors. Furthermore, we use an average grid mix that likely overestimates electric bus impacts, since their charging could be managed and they could also potentially provide ancillary services to the grid.

Although we assess total benefits for the entire population, the benefits of switching to electric school buses would likely be much higher in disadvantaged communities that are overburdened by pollution, helping deliver on the Federal government’s Justice40 initiative (24, 25, 27). Traffic emissions, especially elemental carbon, are key contributors to large disparities in PM_2.5_ air pollution concentrations observed within short distances (≪1-km), which are much higher near highways (28, 53). Replacing old school buses seems particularly beneficial in reducing these disparities even when compared to other vehicle emission controls, because primary PM_2.5_ emissions account for a much larger share of old school bus health impacts relative to other vehicles (4). Traffic emissions are also an important contributor to racial and ethnic disparities in PM_2.5_ air pollution exposure (29). However, these disparities occur on a much finer geographic scale than our model is able to capture (28, 53, 54). In addition to racial and ethnic disparities in exposure, national epidemiologic studies suggest that the health effect per unit PM_2.5_ exposure is roughly 50% larger among Hispanic Americans, 2 to 3 times as large among Black Americans, and 2 times as large for low-income Americans, which suggests that Black, Hispanic, and low-income Americans derive substantially larger health benefits if ambient PM_2.5_ air pollution is reduced (55, 56).

An important limitation of our study is that we do not assess how electric buses might reduce children’s exposure to in-cabin air pollution while riding buses. A better understanding of this additional health benefit is particularly important for policy decisions surrounding electric bus deployment in rural areas, where health benefits attributable to lower ambient PM_2.5_ exposure are small and insufficient to make electric buses cost-beneficial. Studies have reported that adoption of emission controls and low-emission school buses improves lung function, especially in children with asthma (57), reduces hospitalizations (58), and improves attendance (57, 59). However, these effects were observed in very old and polluting buses with few emission controls, and it is uncertain whether they apply to the current diesel bus fleet. Studies of pre-MY 2007 school buses have suggested that self-pollution contributed an average of 7 to 8 μg/m^3^ to in-cabin PM_2.5_ concentrations (60–62), an exposure as large as current average ambient PM_2.5_ levels in the United States (7.8 μg/m^3^) (63). However, 80% of this PM_2.5_ self-pollution was estimated to come from crankcase emissions (61, 62), which were allowed to be released unfiltered until MY 2007 (64–66). Uncertainty remains about self-pollution in newer buses, as well as about the share of older buses in the current fleet that have been retrofitted. Furthermore, little is known about the toxicity of crankcase PM_2.5_ relative to that of tailpipe or of the ambient PM_2.5_ mix. This results in large uncertainty about risks of in-cabin exposure in the current school bus fleet, and additional exposure assessment studies to reduce this uncertainty would be valuable to inform policy decisions, as well as studies exploring the toxicity of crankcase PM_2.5_.

Effects of in-cabin exposure on school attendance would have large economic impacts, since each school day missed results in $600 to $1,000 in lost learning and productivity (67). A recent study assessed attendance benefits of EPA’s School Bus Rebate Program but found significant effects only when pre-MY 2000 buses are replaced, with null values for newer buses (59). As pre-MY 2000 buses represent only about 3% of the current fleet (13), it is uncertain how much electric bus adoption would improve attendance. Furthermore, adopting high efficiency filters in diesel buses is another alternative that could substantially reduce in-cabin exposure (68).

Another important limitation is that we do not consider the impacts of emissions along the bus life cycle other than GHG emissions associated with the electric bus battery (69). A recent life cycle assessment study of light-duty EVs in China suggests that GHG emissions from vehicle production are substantially smaller than those from vehicle use and that differences in GHG emissions between electric and combustion vehicle production are small (70). In terms of health impacts, use-phase emissions of diesel school buses driven in densely populated areas cause higher exposure and health impact per mass emitted than other life cycle emissions that occur in less densely populated areas; however, there are important environmental justice questions as some populations face only harms from life cycle emissions, e.g., from power plants or mining operations, while not deriving any benefits from improved urban air quality (71–74). Furthermore, there are other types of environmental impacts along the vehicle life cycle that are not captured in our analysis (19, 72, 73).

### Uncertainty in Our Results.

Uncertainty in our results comes primarily from the uncertainty about the relationship between PM_2.5_ exposure and mortality in the case of health impacts, and from uncertainty about the social cost of CO_2_ in the case of climate impacts. Monetized health impacts for each outcome (mortality and childhood asthma) can be roughly approximated as a multiplicative form $\tilde{\beta }\times \tilde{\mathrm{iF}}\times \tilde{\mathrm{EF}}\times \tilde{M_{0}}\times \tilde{V}$, where $\tilde{\beta }$ is the “slope” of the concentration–response function (i.e., % increase in mortality or incidence of childhood asthma per each 1 μg/m^3^ increase in ambient PM_2.5_), $\tilde{\mathrm{iF}}$ is the pseudo intake fraction reflecting the marginal change in ambient concentration per unit of emission (dC/dE), $\tilde{\mathrm{EF}}$ is the emission factor, $\tilde{M_{0}}$ is the baseline mortality or incidence of childhood asthma, and $\tilde{V}$ is the value per statistical life (VSL) or per statistical case (VSC) of childhood asthma. Mortality risk reductions represent over 90% of our monetized impacts and therefore uncertainty about our overall results comes primarily from the uncertainty about these benefits. While all terms are uncertain, uncertainty about $\tilde{\beta }$ is larger than about $\tilde{\mathrm{iF}}$, $\tilde{\mathrm{EF}}$, $\tilde{M_{0}}$, and $\tilde{\mathrm{VSL}}$. Epidemiologic studies and important syntheses of evidence find slopes $\tilde{\beta }$ that can vary within a factor of 2 to 3, with the Global Exposure Mortality Model (GEMM) (8), which we use, representing roughly the median value (4, 8, 75, 76). However, these studies treat all ambient PM_2.5_ as equally toxic by mass and therefore do not capture possible differential toxicity, which substantially increases the uncertainty about $\tilde{\beta }$ in our study.

While the evidence is not conclusive, carbonaceous particles such as primary PM_2.5_ from diesel engines could be much more toxic than the ambient mix by mass. If they were 5 times more toxic, consistent with an older structured expert judgment study by Cooke and colleagues (77, 78), impacts and benefits of replacing old diesel buses in metropolitan areas would be roughly 3 times as large. More recent analyses suggest carbonaceous particles could be even more toxic. In the American Cancer Society Cancer Prevention Study-II cohort, Thurston et al. found that PM_2.5_ from coal combustion and elemental carbon were 5 times and 10 times more toxic by mass than the ambient mix for ischemic heart disease mortality, respectively, though with large uncertainty (79). In more recent analyses of the Medicare cohort using newly developed fine-scale exposure models with 50-m resolution in urban areas (53), black carbon was found to be roughly 10 to 20 times more toxic by mass than the ambient mix for all-cause mortality (80) as well as for incidence of dementia and Alzheimer’s disease (81). The possibility that primary PM_2.5_ from diesel engines is substantially more toxic than the ambient mix suggests that benefits of electrification could be larger and that it might be cost-beneficial to replace a larger share of the diesel buses in fleet than our base results indicate. In the absence of conclusive evidence, a structured expert judgment study would be particularly helpful to inform decisions in a timely manner (77, 82–85). We do not include primary PM_2.5_ emissions from power plants, but, as they represented only a small (11%) share of the total power plant health impacts in 2014 (86), our results would not substantially change even if primary particles from coal combustion are also more toxic than average.

The uncertainty about $\tilde{\beta }$ is much larger than the uncertainty about InMAP’s results ($\tilde{\mathrm{iF}}$) for older buses in large metropolitan areas because most of their impacts come from primary PM_2.5_ emissions. InMAP’s performance varies substantially by pollutant: It performs best for primary PM_2.5_ emissions, for which uncertainty in $\tilde{\mathrm{iF}}$ is small, and worse for NO_x_ and NH_3_ emissions, for which uncertainty in $\tilde{\mathrm{iF}}$ much larger due to nonlinearities in particulate nitrate and ammonium formation (87). Although it is not currently possible to measure changes in ambient concentrations as a consequence in marginal changes in emissions, Tessum et al. compare InMAP with WRF-Chem for 11 diverse emission scenarios (87) and show near-perfect agreement for primary PM_2.5_ emissions: population-weighted R^2^ = 0.99, mean fractional bias (MFB) = −10%, mean fractional error (MFE) = 13%. Performance is substantially worse for nitrates: R^2^ = 0.47, MFB = 69%, MFE = 140%, although Tessum et al. note that this poor performance is driven by locations with very low nitrate concentrations, which may be less consequential for policy and have less influence on total ambient PM_2.5_ concentrations (87). Although InMAP was designed to estimate the effects of marginal changes in emissions, not total ambient PM_2.5_ concentrations, it still achieved an MFB of −6% and an MFE or 36% against total observed PM_2.5_ concentrations in 840 monitoring sites in 2011—which accounts for uncertainty in InMAP and in its inputs, including emissions inventories and meteorology (2). InMAP’s low bias when predicting total ambient PM_2.5_ concentrations nationwide suggests that uncertainty about $\tilde{\mathrm{iF}}$ is reduced when many counties are aggregated, such as our results aggregating counties by degree of urbanization.

Uncertainty about $\tilde{\mathrm{iF}}$ varies by school bus MY and driving location, because these two factors determine how much of the total impacts come from primary PM_2.5_ emissions. Uncertainty about $\tilde{\mathrm{iF}}$ is smaller for old buses in large metropolitan areas, where 50 to 60% of estimated health impacts comes from primary PM_2.5_ emissions, and larger for newer buses and those driven in low-population density areas, where NO_x_ accounts for a vast majority of impacts. However, because benefits are very small when replacing newer buses or those driven in low population-density areas, it seems unlikely that this uncertainty would substantially change our conclusion—especially since a high share of NO_x_ suggests the PM_2.5_ from these buses might be less toxic by mass.

Uncertainty about the $\tilde{\mathrm{VSL}}$, while smaller, also contributes to uncertainty in our results. We use a VSL of $12.4 million (2022 dollars and income levels), updated from 2014 estimates reported in U.S. Department of Health and Human Services guidance (88, 89) (*Materials and Methods*); that guidance recommends sensitivity analysis within roughly ±50% of the mean (88). Our results would scale proportionally with a different VSL.

Uncertainty about our estimates of the value of reducing childhood asthma is likely much larger in a relative sense, but this uncertainty would not substantially affect our conclusions as asthma contributes less than 10% of overall monetized impacts. There is relatively little empirical research that estimates the value of reducing the risks of asthma incidence. We use a VSC of $610,000 from a recent willingness to pay study (90) and our asthma impacts would be roughly 10 times smaller if we used EPA’s (67) valuation approach, which yields $45,000 (2015 USD) per attributable case of childhood asthma following a cost of illness study that includes only lifetime healthcare costs and productivity losses (91).

Uncertainty about $\tilde{\beta }$ and $\tilde{M_{0}}$ for childhood asthma are also large. We use $\tilde{\beta }$ from EPA’s (67) recommended concentration–response function, from a Canadian cohort (92) (β = 4.4% per 1 μg/m^3^), which is about 50% larger than mean slopes (β = 2.9% and 3.0% per 1 μg/m^3^) in two important U.S. cohorts (93, 94) identified by the EPA. Furthermore, the 95% CI of β included the null in both of these U.S. cohorts (93, 94). All three studies assume all PM_2.5_ is equally toxic by mass, therefore not capturing potential differential toxicity of diesel bus emissions.

Regarding $\tilde{M_{0}}$, we also use EPA’s (67) recommended age-specific childhood asthma incidence rates, from Winer et al. (95) (12.5/1,000 on average across age groups), which are 2 to 3 times smaller than a recent pooled estimate of nine U.S. birth cohorts (96). Winer et al.’s data are older, from 2006 to 2008, but come from a general population sample covering 24 to 34 states, whereas most cohorts in Johnson et al. (96) recruited children at higher risks of developing asthma and include data from 1980 to 2017 from eight states. Johnson et al. estimate that incidence rates increased over time and peaked at 41.9/1,000 from 2005 to 2009 when data from all cohorts are pooled. Their incidence rates among children without parental history of asthma (17.5/1,000) and among the general risk population-based cohorts (varying from 14.2/1,000 to 31.2/1,000) are still somewhat higher than Winer et al.’s.

Uncertainty in our climate impacts comes largely from uncertainty about the social cost of CO_2_. We use EPA (97, 98) estimates of $228 per metric ton in 2023 (2022 USD), which are similar to the values of Rennert et al. (99), whose work is one of the three damage functions used by the EPA (97, 98). Rennert et al. estimate a 90% CI of $44 to $413 in 2020 (2020 USD) per metric ton using a 2% discount rate, suggesting that the social cost of CO_2_ could be roughly twice as large or four times as small as the authors’ base value ($185 in 2020, 2020 USD). Our climate impacts and benefits would scale accordingly. However, even if climate benefits were twice as large, they would still account for only about half of the average $156,000 cost differential between electric and diesel school buses and health benefits would still be crucial in determining whether the benefits of replacement exceed the costs.

## Conclusion

Electric school bus adoption would lead to health benefits due to reduced exposure to ambient air pollution as well as the reduced GHG emissions. Replacing old diesel buses with electric school buses in large metropolitan areas achieves very large health benefits which accrue largely to the local population, and similarly large health benefits are achieved when the old diesel buses in large metropolitan areas are replaced by new low-emission buses. Policies that accelerate fleet turnover in densely populated areas deserve consideration and are likely cost-beneficial in many areas. Some areas derive much larger health benefits than others, and determining which strategy is most cost-beneficial in each district requires combining our estimates with a thorough analysis of location-specific costs. More research is needed to understand the health benefits of electric school buses to children riding the buses. This is especially important to inform policy in rural areas, where many of the older diesel buses are driven, causing small impacts on population exposure to ambient PM_2.5_ but potentially large impacts on children riding the buses.

## Materials and Methods

### Diesel School Bus Health Impacts.

To estimate air pollution impacts of diesel school buses, we follow our previously developed methodology (4, 100). It is based on three main components: i) an estimate of the impacts of school bus emissions on ambient PM_2.5_ exposure; ii) concentration–response functions from epidemiologic studies, linking ambient PM_2.5_ concentration to adult mortality and childhood asthma onset risks; and iii) baseline mortality and asthma incidence rates. We then use willingness to pay measures to estimate the monetized value of these impacts. For mortality, we apply our previously estimated marginal impacts on mortality per mass emitted of PM_2.5_, SO_2_, NO_x_, NH_3_, and VOCs in each U.S. county in 2017 to diesel school bus emissions of each pollutant, adjusting monetized values to 2022 dollars and income levels. For childhood asthma risks, we calculate new marginal impacts as these were not covered in our previous estimates.

Regulatory analyses by the U.S. EPA have suggested that over 90% of the monetized benefits of air quality standards are due to reduced adult mortality attributable to chronic PM_2.5_ exposure, even as PM_2.5_ is also known to cause a variety of nonfatal effects with higher incidence than mortality (e.g., asthma exacerbations, hospital admissions, lost school and work days, myocardial infarctions) (101, 102). We also account for new childhood asthma cases because of recent evidence suggesting large parental willingness to pay to reduce childhood asthma risks in the United States (90).

For the first component, we characterize emissions of the fleet in 2017 using a set of previously developed county-level real-world emission factors for school buses (4) using data from EPA’s 2017 NEI (44) (*SI Appendix*, Table S1). We adjust these emission factors to remove the tire and brake wear portion, whose impacts are small and likely similar in diesel and electric buses (*SI Appendix*, section 1.4). For MYs 2005, 2010, and 2020, we use U.S. average lifetime average emission factors per mile from the GREET model (12), which we apply to diesel buses driving in all locations (*SI Appendix*, Table S1).

We then calculate changes in ambient PM_2.5_ concentrations as a consequence of school bus emissions using the InMAP air pollution model’s source-receptor matrix (ISRM) (2, 87, 103). The ISRM divides the contiguous United States in 52,411 cells of variable size and provides estimates of marginal changes in PM_2.5_ concentrations in each receptor cell as a consequence of marginal changes in emissions of each pollutant in each source cell. ISRM cells are smaller in more densely populated areas, where they are as small as 1 x 1 km. We use our previous mapping of ISRM cells to counties in the contiguous United States weighting by population, which uses population on a Census block level and assumes that the within-county spatial distribution of school bus emissions follows that of population (4, 100).

For the second component, for childhood asthma we use EPA’s recommended concentration–response function for benefits analysis (67), from a Canadian cohort studying the relationship between long-term PM_2.5_ exposure and asthma onset among children aged 0 to 12 y (92). We use effect estimates from Tétreault et al.’s (92) adjusted model with time-varying exposures, which yielded a hazard ratio of 1.33 per IQR of exposure (6.53 μg/m^3^). We implement it as a log–linear concentration–response function, yielding a 4.4% increase in asthma risk per each 1 μg/m^3^ increase in ambient PM_2.5_, as Tétreault et al. found that nonlinear models did not significantly improve model fit (92). We further assume a no-threshold model, since the minimum exposure in this Canadian cohort (2.32 μg/m^3^) was much lower than typical U.S. levels. We apply this concentration–response function to all children under 17 y old, following EPA’s recommendation based on the similarity of physiology and etiology of lung development in children between 6 and 17 y old (67).

For mortality, we use our previously calculated marginal damages (4) using age-specific concentration–response functions linking ambient PM_2.5_ exposure to nonaccidental mortality risks from the GEMM (8) (*SI Appendix*, section 1.6). GEMM was a collaboration among the principal investigators responsible for 15 of the largest epidemiological cohorts studying the relationship between ambient PM_2.5_ and mortality and used individual-level data from these cohorts to fit a unified model. It also included summary statistics for another 26 cohorts (8).

For the third component, we estimate incidence of childhood asthma by county using age-specific national-level incidence and prevalence rates, and county-level age-specific population (*SI Appendix*, section 1.5). We apply national-level asthma incidence rates estimated by Winer et al. (95), using data from the Behavioral Risk Factor Surveillance System Asthma Call-Back Survey, to at-risk children in each county, which we define as children not currently with asthma. We estimate the number of children of each age group not currently with asthma in each county using age-specific county-level population in 2019 (104) and national-level prevalence asthma rates in 2019 from the National Health Interview Survey (105). For mortality, our previously estimated values (4) applied county-level baseline age-specific mortality rates for the period between 2014 and 2018 from the U.S. CDC (106) to county-level age-specific population counts in 2017 from HHS (107).

Marginal impacts per mass emitted of each pollutant are assessed with equations S1-S2 (*SI Appendix*, section 1.6), following our previous work (4). We estimate the economic value of attributable new childhood asthma cases using a VSC of $610,000 (2022 USD), the U.S. value—elicited from a sample of U.S. parents of nonasthmatic children—from a recent discrete choice experiment surveying residents of seven countries (90). For mortality risks, our previous work estimated impacts in 2017, therefore we adjust the results to 2022 dollars and income levels by updating the VSL. We start with the U.S. Department of Health and Human Services recommended VSL of $9.3M in 2014 (88, 89), adjusting for real income growth using median usual weekly earnings (108) and an income elasticity of 1.0, and adjusting for inflation using the U.S. GDP deflator (109). This results in a VSL of $12.4 million per attributable death (2022 dollars and income levels).

We further assume there is a cessation lag between changes in PM_2.5_ exposure and changes in mortality and apply the preferred cessation lag structure recommended by the EPA (110). This structure assumes that 30% of the benefits occur in the first year, 50% occur uniformly between years 2 and 5, and the remaining 20% occur uniformly between years 6 and 20. To reflect time preferences, we discount all health economic benefits applying the commonly used discount rate of 3% per year (111), consistent with the rate used to discount replacement costs. We report all monetized values in 2022 USD and income levels.

Although our model estimates health impacts at the county level, we also estimate impacts for each school district by mapping county-level health impacts to school districts (45, 46), weighting by population at the Census block-group level (112–114) (*SI Appendix*, section 1.7).

### Health Impacts of Electric School Buses.

For electric buses, we apply our previous estimate of the U.S. grid average PM_2.5_-attributable mortality per mass emitted in 2018 (39) to projected grid emissions of NO_x_ and SO_2_ during the bus lifetime using U.S. EIA’s reference-case projections of emissions for the next 13.5 y, from 2023 to 2036 (51) (*SI Appendix*, section 1.2). We assume electric school buses consume 1.54 kWh per mile driven (13, 30), in addition to 4.8% electricity grid losses (115) and 10% charging losses (116) (*SI Appendix*, section 1.3). We estimated the monetized value of these impacts using the same VSL, cessation lag, and discounting used for diesel school bus emissions.

To account for impacts on childhood asthma onset, we assume that the ratio of asthma to mortality impacts from power plant emissions is the same as our calculated ratio for diesel bus emissions. This assumption is consistent with approximating impacts (*SI Appendix*, Eq. **S1**) as β x ΔC x M_0_. Although diesel bus and electricity emissions occur in different locations and the slope (β) and baseline mortality (M_0_) vary spatially, the error introduced is small since we assess the average impacts of the entire U.S. grid, covering emissions spread throughout the entire contiguous United States.

### Sensitivity Analysis—Differential Toxicity of PM_2.5_.

The previous calculations assume that all PM_2.5_ is equally toxic by mass, regardless of its source or composition. While the evidence is not conclusive, recent studies suggest that carbonaceous particles from coal combustion and traffic are substantially more toxic by mass than the ambient mix (79–81). To reflect this possibility, we conduct a sensitivity analysis assuming primary PM_2.5_ emissions from combustion sources (in our case, primary PM_2.5_ emissions from diesel buses) are 5 times more toxic than the ambient mix by mass, which follows the risk assessment by Lelieveld et al. (78) and is consistent with the previous expert judgment by Cooke et al. (77).

### Climate Impacts.

We complement our estimates of air pollution impacts with an estimate of climate benefits covering GHG emissions from diesel bus tailpipes and from electricity generation and battery production in the case of electric buses. For diesel buses, we assume a fleet-average fuel economy of 7.36 miles per gallon (*SI Appendix*, section 1.4), which yields 1,383 grams of CO_2_ per mile assuming 10,180 grams of CO_2_ per gallon of diesel (117).

For electric buses, reference-case projections by the U.S. EIA yield an average of 492 lb. of CO_2_ per MWh for the 13.5-y period between 2023 and 2036 (51, 118). This results in 381 grams of CO_2_ per mile, assuming a consumption of 1.54 kWh per mile (13, 30), 4.8% grid losses (115), and 10% charging losses (116). For battery production, we use a recent estimate of 59.5 kg of life-cycle CO_2_-eq. emissions per kWh of battery (69), yielding 9.9 t of CO_2_ for a 166 kWh battery (13, 30) (*SI Appendix*, section 1.3). We allocate these emissions to the first year; dividing them by the bus lifetime would yield 52 grams of CO_2_-eq. per mile driven.

We estimate the economic value of CO_2_ emissions applying EPA’s estimates for the social cost of CO_2_ that use a near-term discount rate of 2% (97, 98). EPA’s estimates increase over time, from $228.10 (2022 USD) per metric ton for emissions occurring in 2023 to $281.77 (2022 USD) (undiscounted) for emissions occurring in 2036. We apply these values to emissions occurring during the electric and diesel bus lifetimes. Consistent with EPA’s recommendations, we discount the value of future emissions by 2% per year (97).

### Model Implementation.

Our model was implemented in R version 3.5.1 (119) using R packages “ncdf4” (120), “mgcv” (121, 122), and “nlme” (123, 124). Figures and tables were generated with R version 4.3.2 (125), using packages “raster” (126), “sp” (127–129), “viridis” (130), and “TAM” (131).

## Supplementary Material

Appendix 01 (PDF)

Dataset S01 (CSV)

Dataset S02 (CSV)

Dataset S03 (CSV)

Dataset S04 (CSV)

Dataset S05 (CSV)

Dataset S06 (CSV)

## Data Availability

Publicly available and previously published data were used for this work (13, 20, 43–45, 47, 51, 52, 100, 103–109, 112–115, 118). We also use code publicly available from ref. 100. Supplementary results are available in Datasets S1–S6; a description of these datasets is provided in the *SI Appendix*. Additional computer code used and data have been deposited in Harvard Dataverse (https://doi.org/10.7910/DVN/SETWX7) (132).
