# Supplementary material for: Adopting electric school buses in the United States: Health and climate benefits
Source: Proc Natl Acad Sci U S A. 2024 May 20;121(22):e2320338121. doi: 10.1073/pnas.2320338121 (PMC11145267; doi:10.1073/pnas.2320338121)
Supplement: Supplementary file 1 — Appendix 01 (PDF) [file pnas.2320338121.sapp.pdf]

PNAS

Supporting Information for
Adopting electric school buses in the United States: health and climate benefits

Ernani F. Choma[a *], Lisa A. Robinson[b], Kari C. Nadeau[a *]
[a] Department of Environmental Health, Harvard T.H. Chan School of Public Health, Boston MA 02115
[b] Center for Health Decision Science, Harvard T.H. Chan School of Public Health, Boston MA 02115

*Corresponding Authors:
Ernani F. Choma
Email: echoma@hsph.harvard.edu

Kari C. Nadeau
Email: knadeau@hsph.harvard.edu

This PDF file includes:

Supporting text
Figures S1 to S11
Tables S1 to S6
SI References

Supporting Information Text

This supplementary information is organized in two sections. In section 1, we provide additional information on the data and methods used to estimate health and climate impacts. In section 2, we presented supplementary results referred to in the main text. Section 3 contains the descriptions of supplementary datasets S1-S6 (separate files).

1. Data inputs to calculate health impacts

This section describes school bus emissions (sections 1.1 and 1.2), energy consumption (section 1.3), tire and break wear emissions (section 1.4), baseline asthma incidence (section 1.5), the calculation of attributable cases and their monetized value (section 1.6) and the mapping of county-level impacts to school districts (section 1.7).

1.1. Diesel school bus emissions of air pollutants. Emission factors for the fleet in circulation in 2017, as well as for diesel school buses of model years (MYs) 2005, 2010, and 2010 are shown in Table S1. Emission factors for the fleet in 2017 were calculated by Choma et al. (1) using data from the EPA's 2017 National Emissions Inventory (2). They were calculated as total school bus emissions of each pollutant in 2017 in each county divided by total school bus miles travelled in each county in 2017. We further adjust these emission factors to remove the tire and brake wear portion (section 1.4). Emission factors for MYs 2005, 2010, and 2020 are U.S. average lifetime average emission factors per mile from the GREET model calculated by Burnham (3) using EPA MOVES3. As Burnham does not provide location-specific emission factors, we apply the same U.S. average emission factors to diesel school buses MYs 2005, 2010, and 2020 driving in all locations.

Table S1. Real-world diesel school bus emission factors per mile for each pollutant, by school bus model year.

Model Year (MY)	$PM_{2.5}$	SO_2	NO_x	NH_3	VOC
Fleet Average in 2017 (Mean and 95% CI)[a]	0.222 (0.062 – 0.492)	0.008 (0.003 – 0.012)	4.209 (1.805 – 8.511)	0.018 (0.004 – 0.025)	0.565 (0.056 – 1.269)
2005[b]	0.513		7.130		1.281
2010[b]	0.042		1.687		0.197
2020[b]	0.011		0.990		0.084

[a] Source: Calculated by Choma et al. (1) using 2017 National Emissions Inventory data from EPA (2). We further adjust emission factors from Choma et al. to remove the tire and brake wear portion of $PM_{2.5}$ removed (see section 1.4). Mean and 95% CI reflect the variation in emission factors by county and are weighted by school bus miles travelled.

[b] Source: Burnham (3). For $PM_{2.5}$, we use only the exhaust emission factors given by the author. For VOCs, we consider exhaust and evaporative emissions. Burnham does not provide SO_2 and NH_3 emission factors.

1.2. Electric school bus emissions of air pollutants and marginal mortality impacts. Electricity grid emissions of SO_2 and NO_x over the bus lifetime and associated mortality impact are shown in Table S2.

Table S2. Marginal impacts from electricity grid emissions.

	2018		Average between 2023 and 2036	
Pollutant	Emission Factor (lb./MWh)[a]	Attributable Deaths (per billion kWh)[b]	Emission Factor (lb./MWh)[c]	Attributable Deaths (per billion kWh)[d]
SO_2	0.693	1.56	0.179	0.40
NO_x	0.632	0.59	0.218	0.21
Total		2.16		0.61

[a] U.S. grid-average emissions using U.S. EPA eGRID data (4), including 4.8% grid losses (see Choma et al. (5)).
[b] Calculated by Choma et al. (5).
[c] Average of projected U.S. grid-average emissions in U.S. EIA's reference-case (6), incorporating grid losses of 4.8%. These averages weight emissions in future years discounting at 3%/year (unweighted averages are 0.171 lb./MWh for SO_2 and 0.210 lb./MWh for NO_x). The year of 2036 is given half weight to reflect an electric bus lifetime of 13.5 years.
[d] Calculated as attributable deaths in 2018 x ($EF_{2023\text{-}2036}/EF_{2018}$), where EF_y is the emission factor in period y.

1.3. School bus energy consumption. We estimate fleet average diesel and electric school bus energy consumption and battery sizes using data for school buses of types A, C, and D from Levinson et al. (7), and the share of each type in the current school bus fleet using data from Lazer et al. (8) (Table S3). For electric school buses, we further assume grid losses of 4.8%, the average grid losses in 2018 in EPA's eGRID data (4), and charging losses of 10% (9). We only use diesel school bus fuel economy to calculate greenhouse-gas emissions. We do not use it to calculate diesel school bus emissions of other air pollutants.

Table S3. Diesel and electric school bus energy consumption.

	Type A	Type C	Type D	Fleet Average
Share of fleet [%][a]	30	51	19	
DSB Fuel Economy [miles per gallon][b]	10.50	6.59	6.32	7.36
ESB Electricity consumption [kWh/mile][b]	1.12	1.98	1.75	1.54
ESB Battery capacity [kWh][b]	119	199	188	166

[a] Source: Calculated from number of school buses of each type given by Lazer et al. (8). We restrict the fleet to these three most common types, assigning California's buses of types 1 and 2 given by the authors to Type A.
[b] Source: Levinson et al. (7) for types A, C, and D. Fleet average calculated as weighted average of type A, C, and D, weighted by their share of the fleet.
DSB: Diesel School Bus. ESB: Electric School Bus.

1.4. Tire and brake wear emissions. We do not include $PM_{2.5}$ emissions from tire and brake wear (TBW), assuming they are similar in diesel school buses and electric school buses. For current school buses, the EPA estimates that 80% of the $PM_{2.5}$ TBW emissions is due to brake wear, with tire wear making up the remaining 20% (10, 11). U.S. EPA MOVES3 applies the same tire wear emissions to all vehicles of the same class (e.g., school buses) regardless of fuel type (e.g., diesel or electric) (11). For brake wear, the U.S. EPA (11) does not provide different emission factors for electric and diesel school buses but suggests that electric vehicles should have lower emissions due to regenerative braking. In this case, we might be underestimating electric school bus benefits if they reduce TBW emissions; however, this would likely be only a small underestimate since TBW emission factors are relatively small.

The emission factors we previously calculated (1) using data from the 2017 National Emissions Inventory (2) include all emissions during vehicle operation, so that we adjust them by subtracting the TBW portion from the total $PM_{2.5}$ emissions. We use estimates of school bus TBW emissions per mile from EPA MOVES2014 – the MOVES version used in 2017 National Emissions

Inventory – which are 0.0132 g/mi of brake wear and 0.0027 g/mi of tire wear, or 0.0159 g/mi of TBW in total (10). GREET emission factors from Burnham (3) are provided separately for exhaust and tire and brake wear, so we include exhaust only.

Although we do not include TBW emissions in our results under the assumption that they are the same in diesel and electric school buses, we estimate that the TBW portion would cause between $1,100 and $1,500 per bus in health impacts, using U.S. EPA MOVES 2014 and MOVES3 TBW emission factors (10, 11) and assuming that TBW particles are equally toxic as the ambient mix by mass (Table S4). This is 30 to 50 times lower than the health impacts per bus for the average diesel school bus in the fleet in 2017 ($45,800) and almost 100 times lower relative to a MY 2005 diesel school bus (Table S5). For new MY 2020 diesel school buses, the TBW portion represents a majority of the $PM_{2.5}$ emitted and causes more impact than exhaust $PM_{2.5}$, since MY 2020 emit relatively low amounts of exhaust $PM_{2.5}$. However, when considering all pollutants, MY 2020 diesel buses cause $7,600 in health impacts (a large majority from NO_x), a figure that is still 5 to 7 times larger than the impacts of TBW emissions. Therefore, even if electric buses were to cause small increases in TBW emissions, this would have a very small impact in our results, even for new MY 2020 buses.

Table S4. Tire and Brake Wear emission factors and associated health impacts.

	Emission Factor [g/mile]	Health Impact – U.S Average	
	$PM_{2.5}$ (TBW)	$PM_{2.5}$ (TBW) [USD/mile]	$PM_{2.5}$ (TBW) [USD/bus]
		USD per mile	USD per bus
MOVES 2014	0.0159[a]	$0.0093	$1,500
MOVES3	0.01224[b]	$0.0072	$1,100

[a] Source: Sum of tire and brake wear emission factors given by U.S. EPA (10).
[b] Source: Sum of tire and brake wear emission factors given by U.S. EPA (11).

Table S5. Exhaust $PM_{2.5}$ emission factors and diesel school bus health impacts.

	Emission Factor [g/mile]	Health Impact – U.S. Average [USD/mile]			
Model Year	$PM_{2.5}$ (Exhaust)	$PM_{2.5}$ (Exhaust)		All Pollutants	
		USD per mile	USD per bus	USD per mile	USD per bus
Fleet Average in 2017	0.222[a]	$0.12	$18,400	$0.29	$45,800
MY 2005	0.513[b]	$0.30	$47,100	$0.61	$96,100
MY 2010	0.042[b]	$0.025	$3,900	$0.10	$15,100
MY 2020	0.011[b]	$0.0065	$1,000	$0.048	$7,600

[a] Source: 2017 National Emissions Inventory data from EPA (2), with the tire and brake wear portion of $PM_{2.5}$ removed.
[b] Source: Emission Factors from Burnham (3).

1.5. Baseline Asthma Incidence. To calculate baseline asthma incidence, we apply national-level age-specific incidence rates from Winer et al. (12) to the population at risk in each age group in each county in the contiguous U.S. (Table S6). Winer et al. estimate 12-month incidence rates using data from 2006 to 2008. Data for 2006 covered 24 states and DC, whereas data for the other two years covered 34 states and DC. We define the population at risk as children without asthma. We determine the population at risk in each county and age group using single-year-of-age population counts from the National Vital Statistics System (13), subtracting the proportion of children estimated to have asthma in 2019, using national-level prevalence rates from the 2019 National Health Interview Survey (NHIS) (14).

Table S6. Parameters used to calculate baseline asthma incidence.

Age group [years]	Asthma Incidence[a]	Asthma Prevalence[b] [%]	Population at risk[c] [%]
0 to 4	23.4/1,000	2.6	97.4
5 to 11	11.1/1,000	9.1	90.9
12 to 17	4.4/1,000	8.25	91.75

[a] Source: Winer et al. (12)
[b] Source: 2019 National Health Interview Survey (14). For children older than 4 years, the 2019 NHIS give prevalence rates of 9.1% for ages 5 to 14 years and 7.4% for ages 15 to 19 years. We apply the 9.1% rate to the 5 to 11 year-old-group in the present study, and the average rate between those two NHIS age groups (8.25%) to the 12 to 17 year-old-group in the present study.
[c] Calculated as 1 – Asthma Prevalence. We then determine the number of children at risk by applying these percentages to single-year-of-age population counts for each county from the National Vital Statistics System (13).

1.6. Calculating attributable cases and monetized health impacts. Marginal impacts per mass emitted of each pollutant are assessed with Eq. S1, following Choma et al. (1), which yields attributable new asthma cases.

$$\text{Marginal Impacts}_{s,p} = \sum_r \sum_a AM_{r,a}\left(\Delta C_{s,r,p}\right) = \sum_r \sum_a \frac{RR_{r,a}\left(C_{s,r,p}+\Delta C_{s,r,p}\right)-RR_{r,a}\left(C_{s,r,p}\right)}{RR_{r,a}\left(C_{s,r,p}\right)} \times M_{r,a} \quad \text{(Eq. S1)}$$

Where (1):
M is the baseline outcome measure (new childhood asthma cases per year);
AM is the attributable outcome measure (new childhood asthma cases per year);
C is the ambient $PM_{2.5}$ concentration;
ΔC is the change in ambient $PM_{2.5}$ concentration; and
RR is the relative risk from the concentration-response function.
The index $p = 1,2,\ldots,5$ represents different pollutants, index a represents different age groups, and indices s and r represent source and receptor cells (52,411 InMAP cells (15, 16), which were mapped to 3,108 counties in (1)). In our analyses of impacts occurring in certain regions (e.g., inside vs. outside each metropolitan area), we sum receptors accordingly (e.g., r inside the metropolitan area of s, and r outside the metropolitan area of s).

Following Choma et al. (1), we implement this calculation computationally using Eq. S2, which incorporates the mapping from ISRM cells to counties. Eq. S2 yields monetized impacts as we multiply attributable cases by a value per statistical case (VSC) of childhood asthma.

$$\mathbf{MI}_p = \mathbf{P} \times \mathbf{ISRM}_p \times \mathbf{P}^T \times \mathbf{M} \times k \times VSC \quad \text{(Eq. S2) (1)}$$

Where:
MI is a matrix where $\mathbf{MI}_{i,j}$ is the monetized value of the marginal impact on new childhood asthma cases occurring in county j=1,2,…,3108 (receptor) as a consequence of 1 metric ton of emissions in county i=1,2,…,3108 (source);
P is a matrix where $\mathbf{P}_{ij}$ is the percentage of the population of county i=1,2,…,3108 that is within InMAP cell j=1,2,…,52411;
ISRM is a matrix where $\mathbf{ISRM}_{ij}$ is the increase in concentration ΔC [μg/m^3] in InMAP cell j=1,2,…,52411 (receptor) as a consequence of emissions of 1 μg/s in InMAP cell i=1,2,…,52411 (source);
M is a diagonal matrix where $\mathbf{M}_{ij}$ is attributable new childhood asthma cases for an increase in 1 μg/m^3 in ambient concentration in county i=j=1,2,…,3108 if i = j and 0 if i ≠ j; $k = 10^{12}/(24 \times 3,600 \times 365)$ represents the conversion from 1 μg/s to 1 metric ton/year; and
VSC=610,000 [2022 USD] is the value per statistical case (17).
The index p=1,2,…,5 represents the pollutants.
M is calculated as $\mathbf{M} = \text{Dg}(\text{diag}(\mathbf{D} \times \mathbf{S}^T))$, in which **D** is a matrix where $\mathbf{D}_{ij}$ is the number of deaths in county i=1,2,…,3108 and age group j=1,2,3; **S** is a matrix where $\mathbf{S}_{ij}$ is the percent increase in

baseline mortality in county i=1,2,…,3108 and age group j=1,2,3 for an increase in ambient concentrations of 1 μg/m^3; diag(**X**) denotes the vector containing the diagonal elements of matrix **X**; and Dg(x) denotes the square matrix where off-diagonal elements are 0 and diagonal elements are the elements of vector x.

We account for mortality impacts using our previous estimates of mortality per mass emitted of each pollutant in each county (1, 18), which were previously calculated using the same method, using different input data necessary to calculate mortality risks: baseline mortality, relative risks from a concentration-response linking exposure to ambient $PM_{2.5}$ to mortality risks, and a value per statistical life (VSL) (as opposed to the childhood asthma VSC). We further adjust these previously calculated impacts to 2022 dollars and income levels by adjusting the VSL (see main manuscript, methods).

In our previous study (1), we developed estimates of attributable mortality and their economic value in 2017 using different concentration-response functions. In the current study, we use the version that used the age-specific concentration-response functions from the Global Exposure Mortality Model (GEMM) (19), which was our base case in Choma et al. (1). Many cohorts studying the relationship between ambient $PM_{2.5}$ exposure and mortality have been conducted to date and GEMM is an important synthesis of this evidence. GEMM was a collaboration among 15 research groups responsible for 15 of the largest cohorts, where the investigators fit the GEMM concentration-response function directly to individual-level data (19). In addition, it also incorporated published effect estimates for another 26 cohorts. GEMM provides different concentration-response functions, and we used version that applies to age-specific non-accidental mortality, and that includes a recent Chinese Male Cohort. GEMM's concentration-response function is nonlinear in ambient $PM_{2.5}$ levels, with steeper slopes at low concentrations. Although we use the age-specific GEMM concentration-response functions, whose slopes vary, the GEMM version that applies to all adult non-accidental mortality yields a slope of 1.05% increase in adult non-accidental mortality per each 1 μg/m^3 increase in ambient $PM_{2.5}$ exposure at the mean U.S. ambient $PM_{2.5}$ concentration.

1.7. Estimating impacts by school district. To help inform policies focusing on specific school districts, we produce estimates of health impacts benefits for each 13,309 local education agencies (school districts) in the contiguous U.S. (20, 21) by taking the school-age-population-weighted average of the county-level impacts. We use (i) the percentage of each school district intersecting each Census Block Group, using 2023 relationship files for local education agencies (school districts) from the National Center of Education Statistics (20); and (ii) the population aged between 5 and 19 years old for each Census Block group, using 5-year estimates from the 2022 American Community Survey from the U.S. Census Bureau (22), which were then aggregated to counties. For the state of Connecticut, both the NCES relationship files and the 2022 ACS population estimates use the new 2022 Census block groups, following the new 2022 county equivalents. In this case, we map these to the pre-2022 county equivalents in – used in our air pollution model – using 2022 county subdivision to 2020 block group relationship files for Connecticut from the U.S. Census Bureau (23) and total population in each Census block group in 2020 from the Decennial Census, from the U.S. Census Bureau (24).

2. Supplementary Results

Figures S1-S11 show our supplementary results.

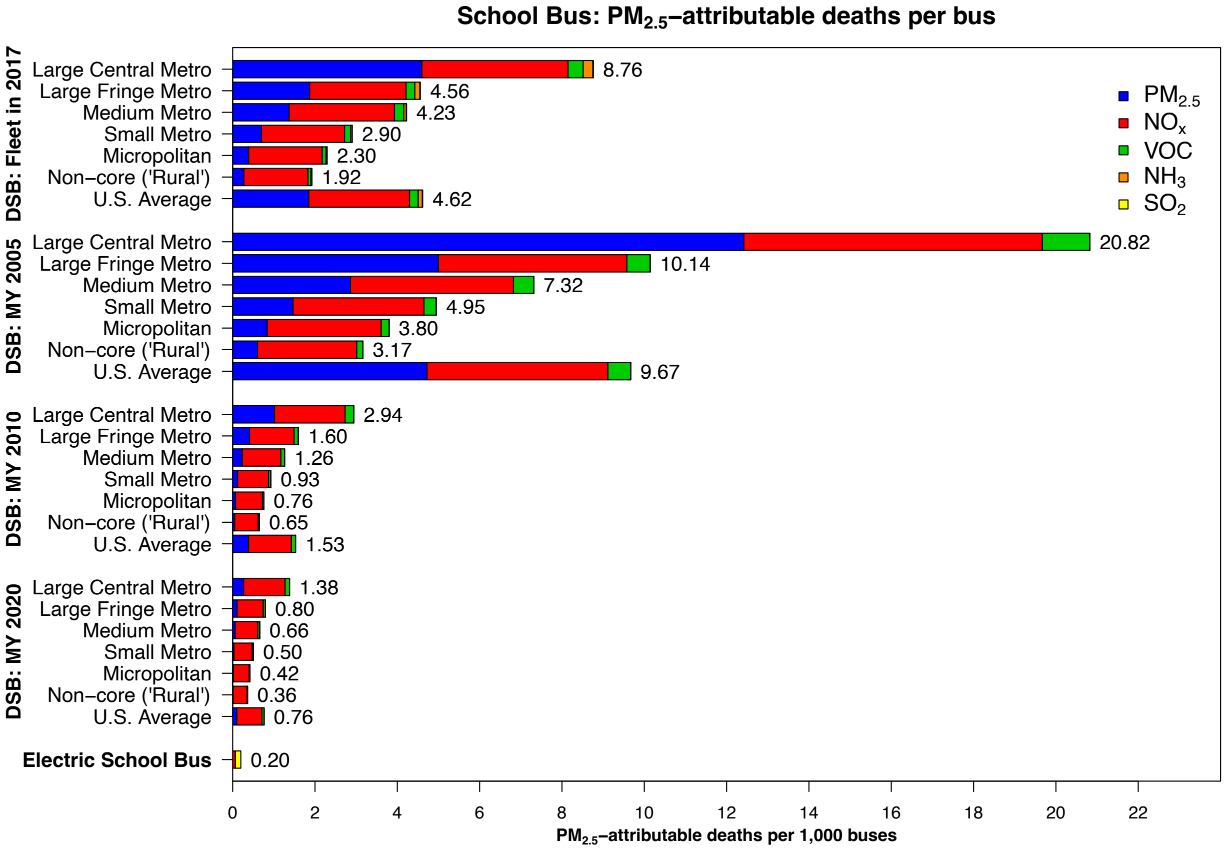

Figure S1. Health impacts of school buses: $PM_{2.5}$-attributable deaths per bus, by bus model year, driving location, pollutant species, and outcome. Locations are classified using NCHS's Urban-Rural classifications (25). DSB: Diesel School Bus. ESB: Electric School Bus.

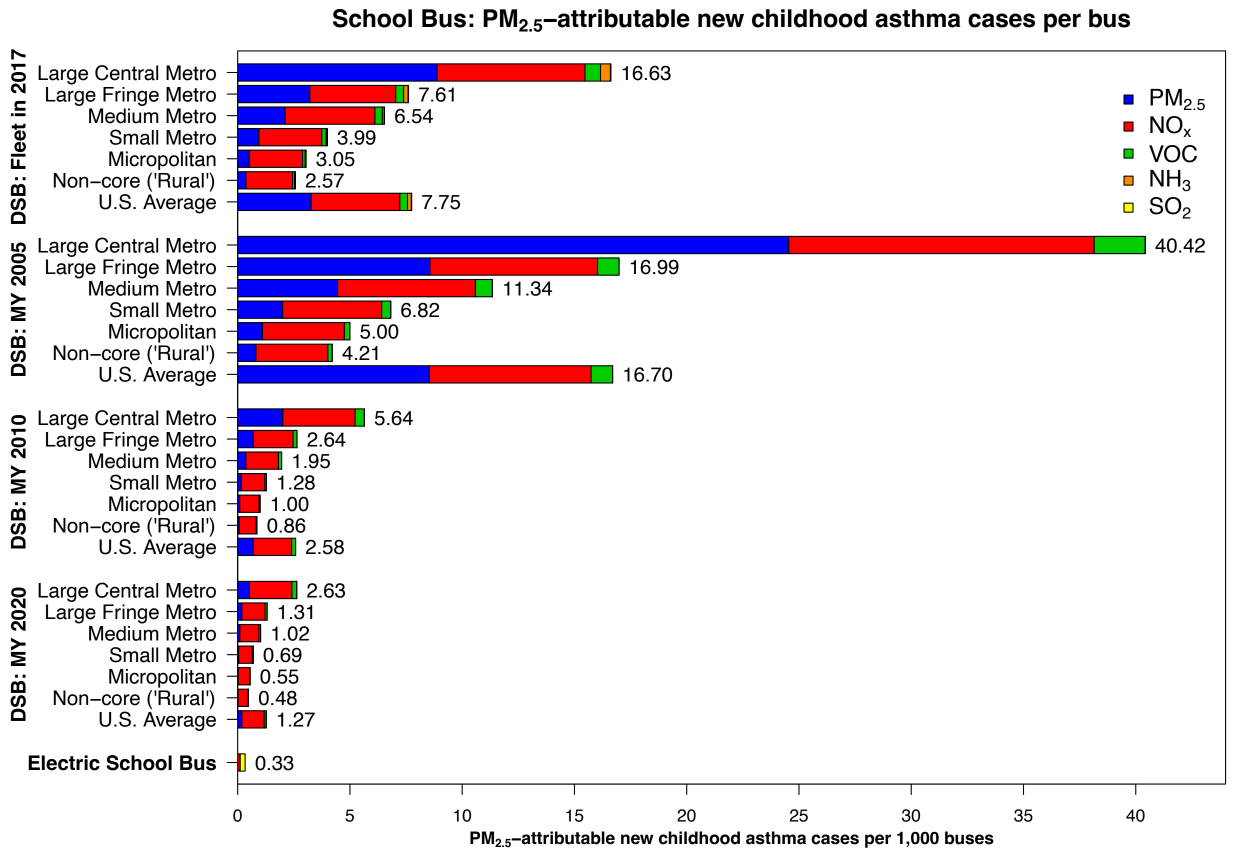

Figure S2. Health impacts of school buses: $PM_{2.5}$-attributable new childhood asthma cases, by bus model year, driving location, pollutant species, and outcome. Locations are classified using NCHS's Urban-Rural classifications (25). DSB: Diesel School Bus.

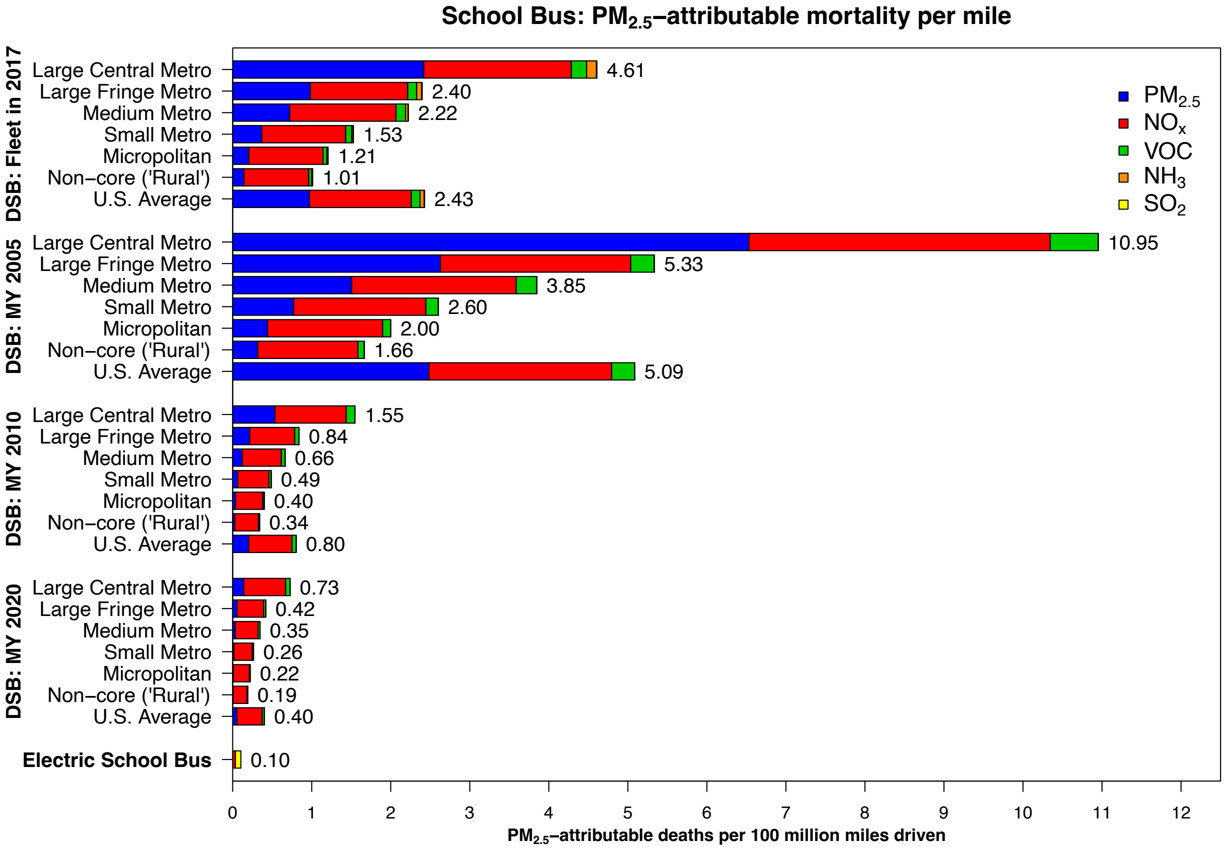

Figure S3. Health impacts of school buses: $PM_{2.5}$-attributable deaths per mile driven, by bus model year, driving location, pollutant species, and outcome. Locations are classified using NCHS's Urban-Rural classifications (25). DSB: Diesel School Bus.

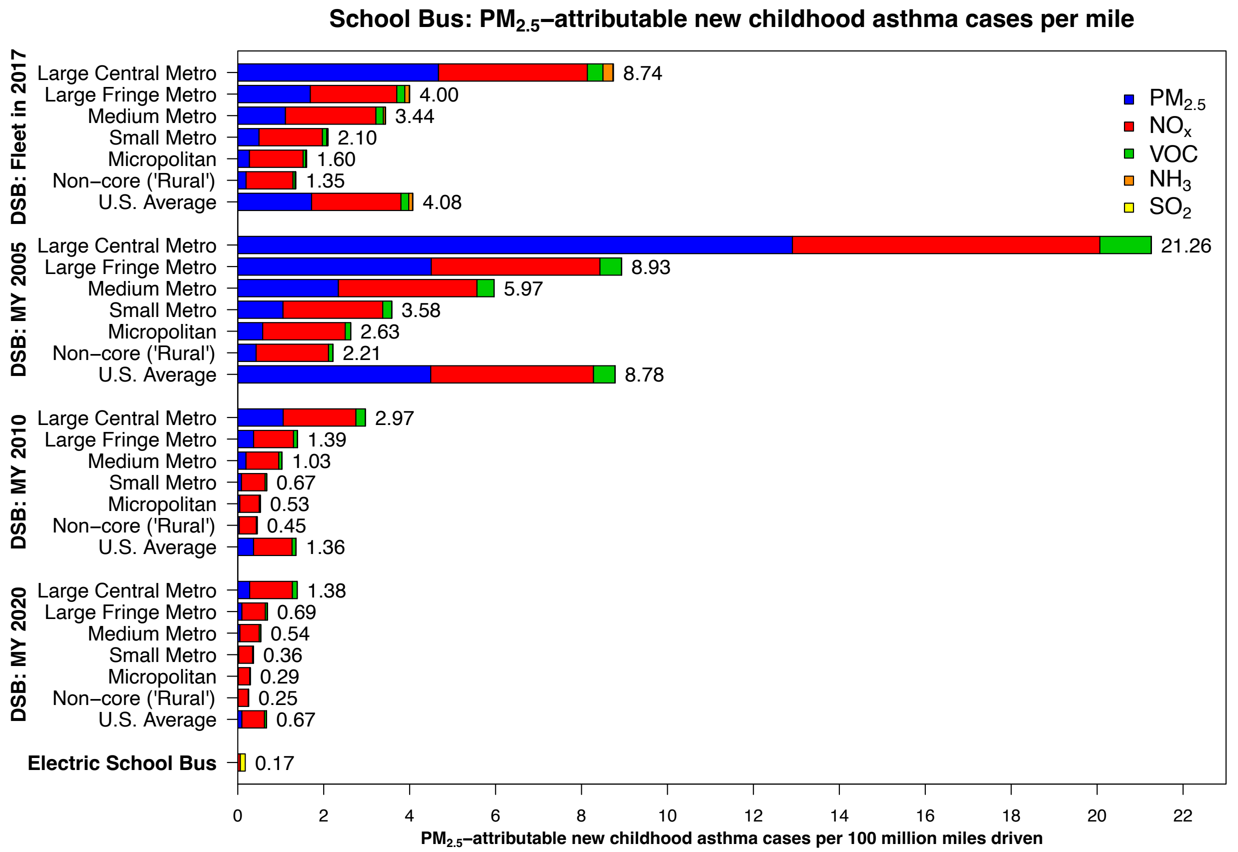

Figure S4. Health impacts of school buses: $PM_{2.5}$-attributable new childhood asthma cases per mile driven, by bus model year, driving location, pollutant species, and outcome. Locations are classified using NCHS's Urban-Rural classifications (25). DSB: Diesel School Bus.

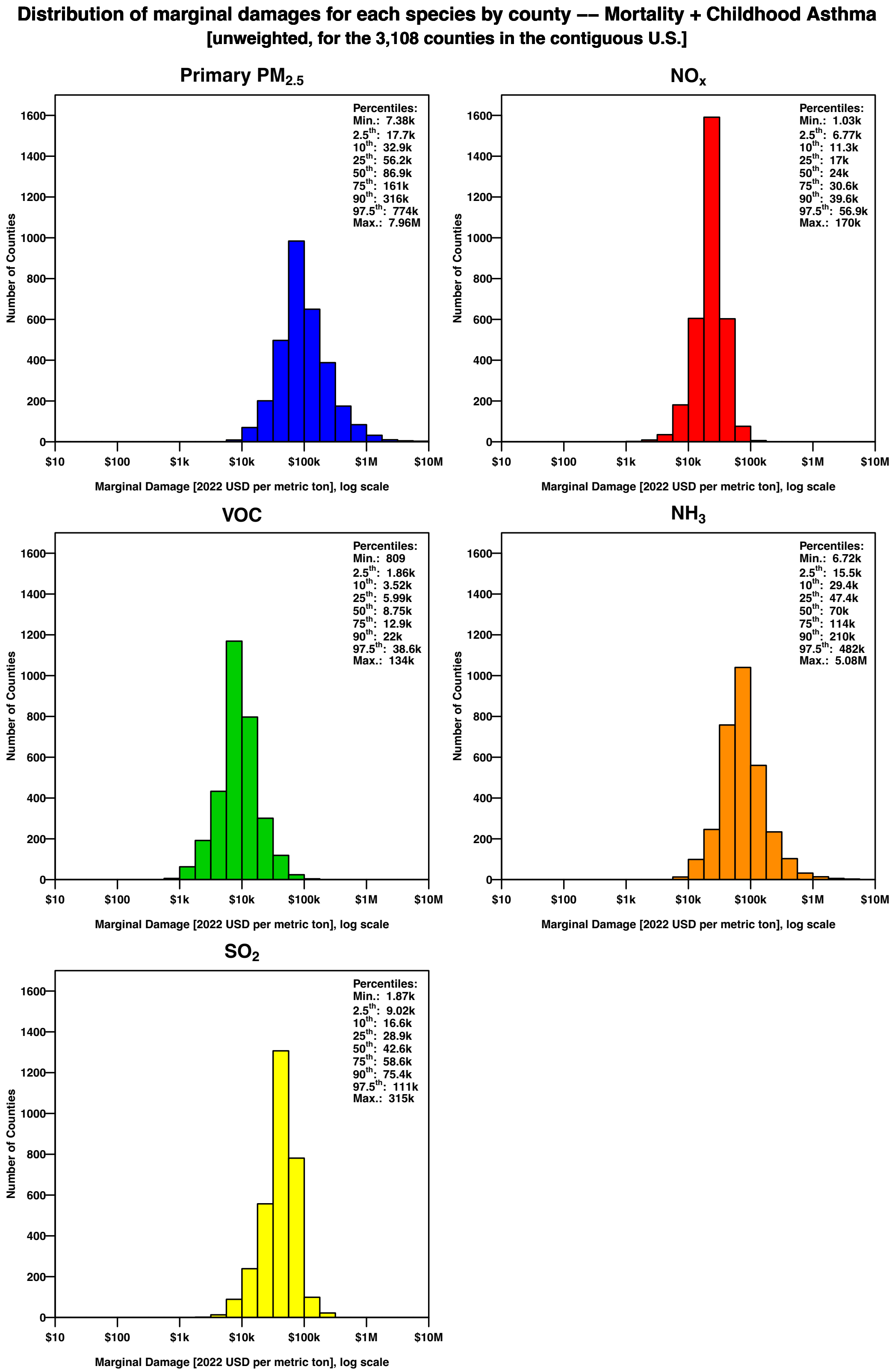

Figure S5. Distributions of marginal damages, including mortality and new childhood asthma cases attributable to $PM_{2.5}$, per ground-level emissions of 1 metric ton of each species in each county.

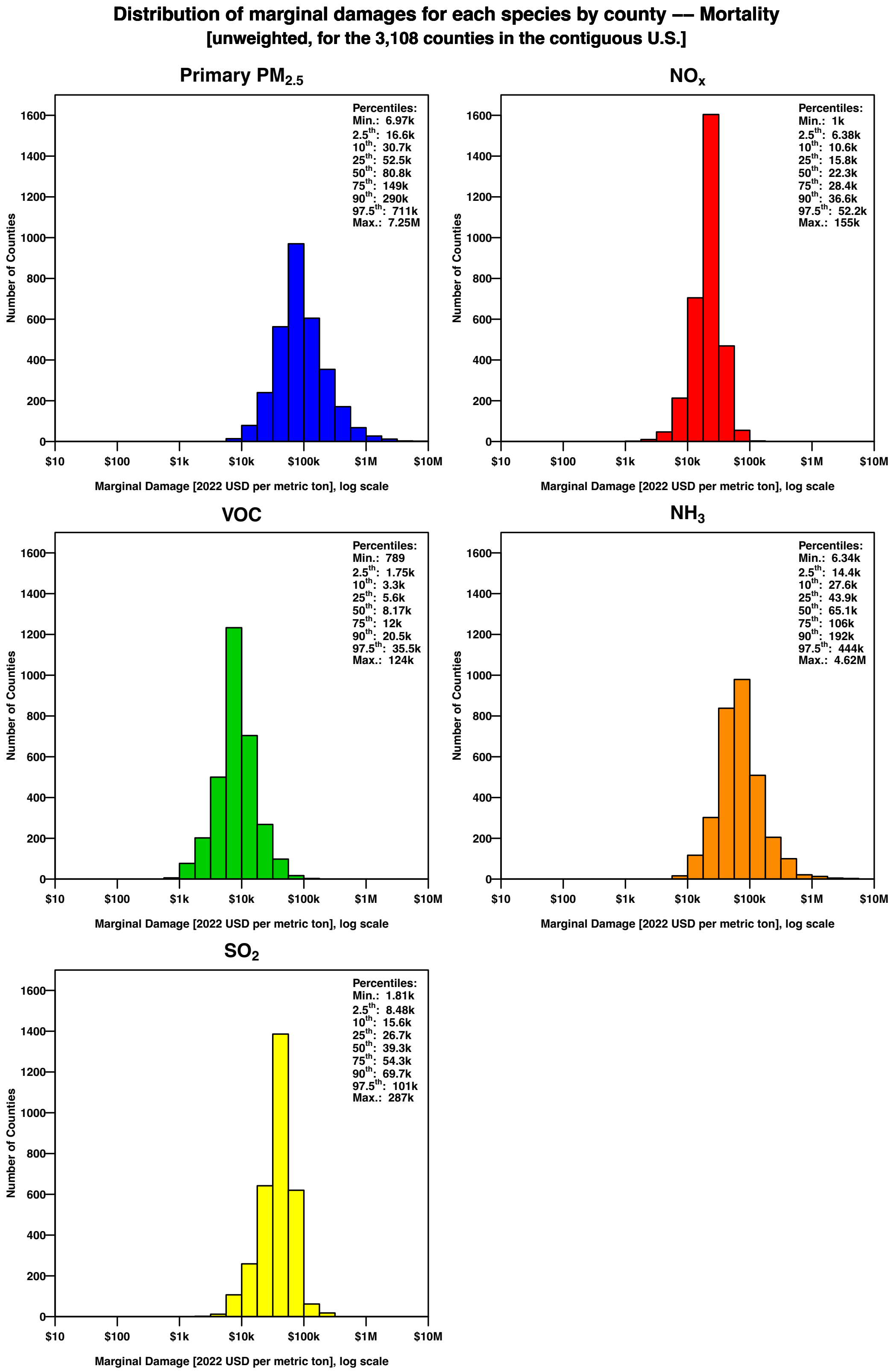

Figure S6. Distributions of marginal damages, including only mortality attributable to $PM_{2.5}$, per ground-level emissions of 1 metric ton of each species in each county. Source: adjusted from Choma et al. (1), adjusting the value per statistical life to 2022 dollars and income levels.

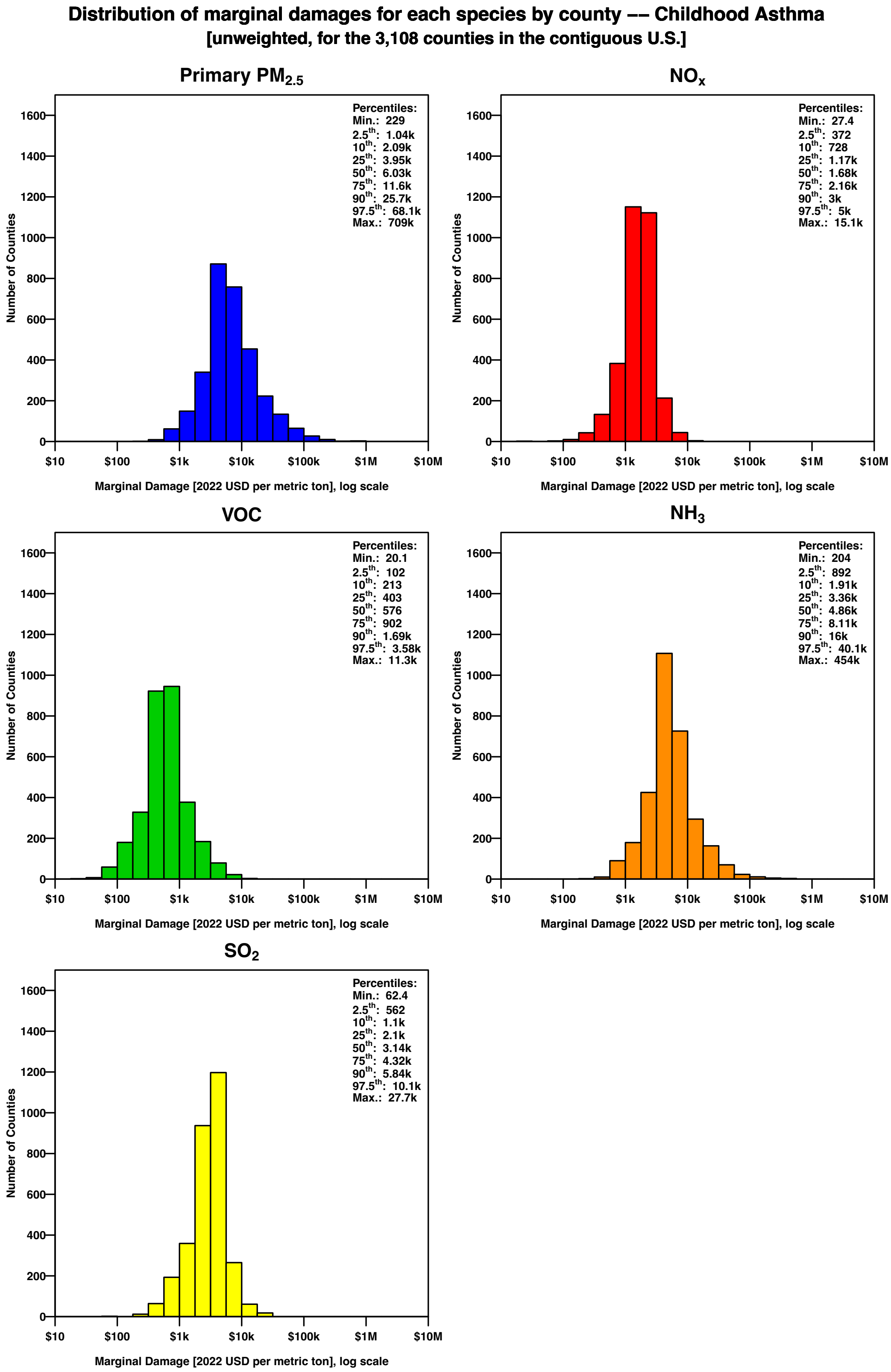

Figure S7. Distributions of marginal damages, including only new childhood asthma cases attributable to $PM_{2.5}$, per ground-level emissions of 1 metric ton of each species in each county.

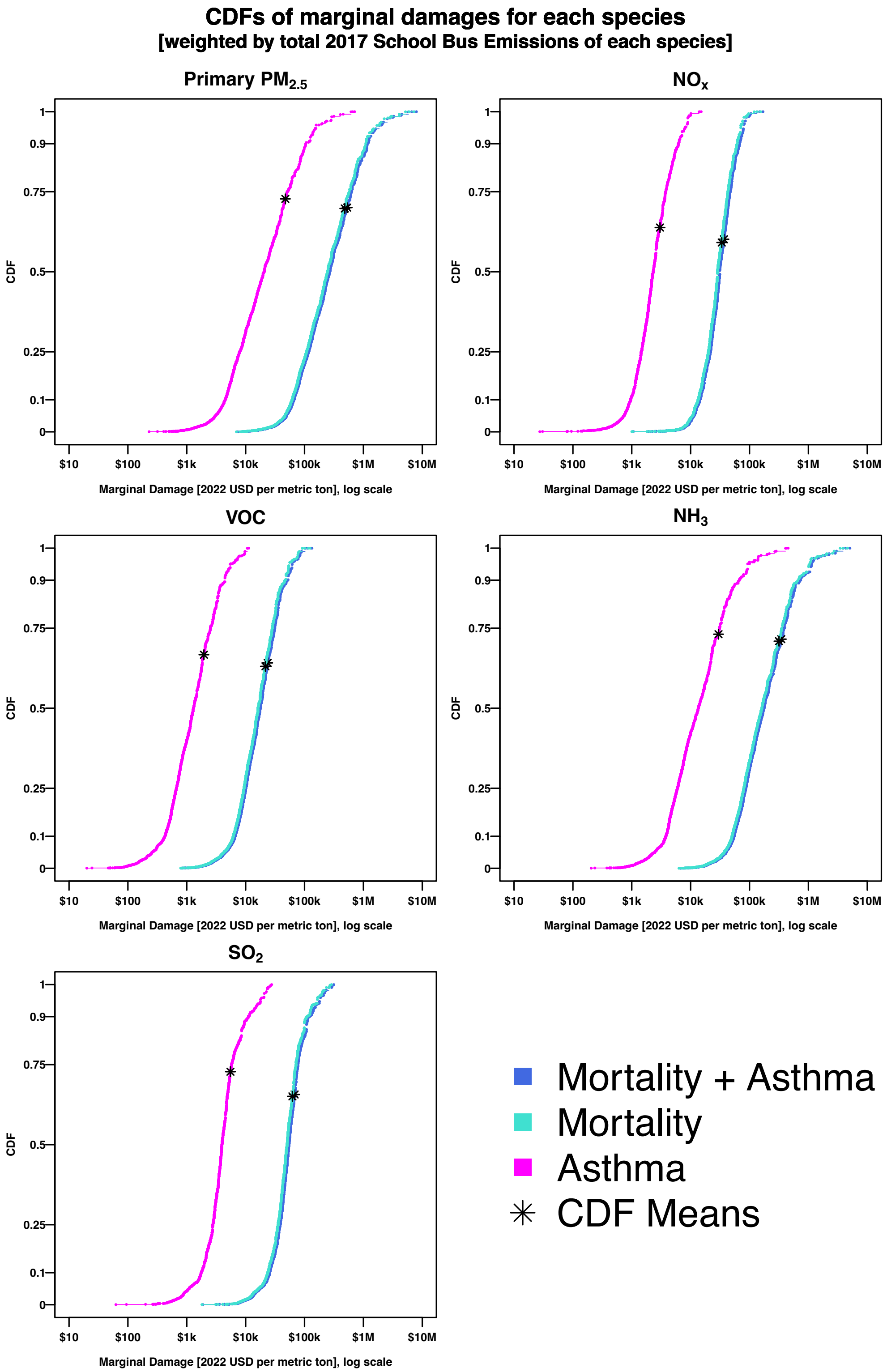

Figure S8. Cumulative distributions of marginal damages, including both mortality and new childhood asthma cases attributable to $PM_{2.5}$, per ground-level emissions of 1 metric ton of each species in each county. Cumulative distributions weighted by total school bus emissions of each species and each county in 2017. Mortality marginal damages are adjusted from Choma et al. (1), adjusted to reflect the value per statistical life in 2022 dollars and 2022 income levels.

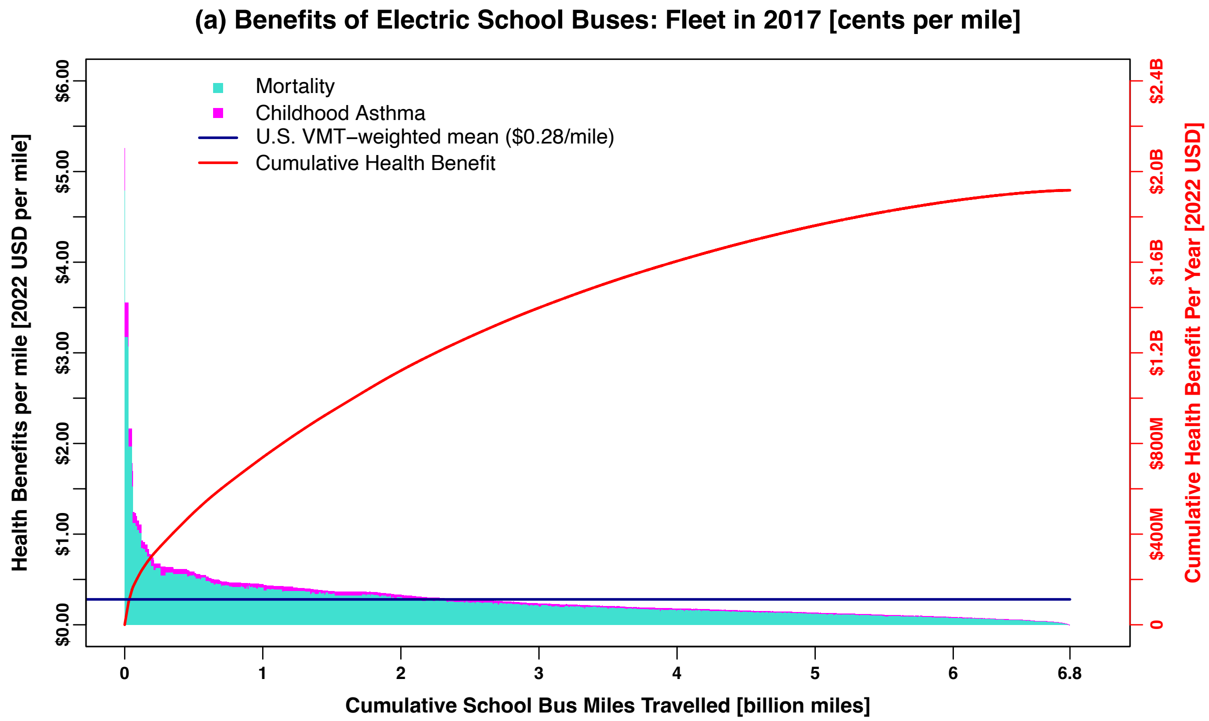

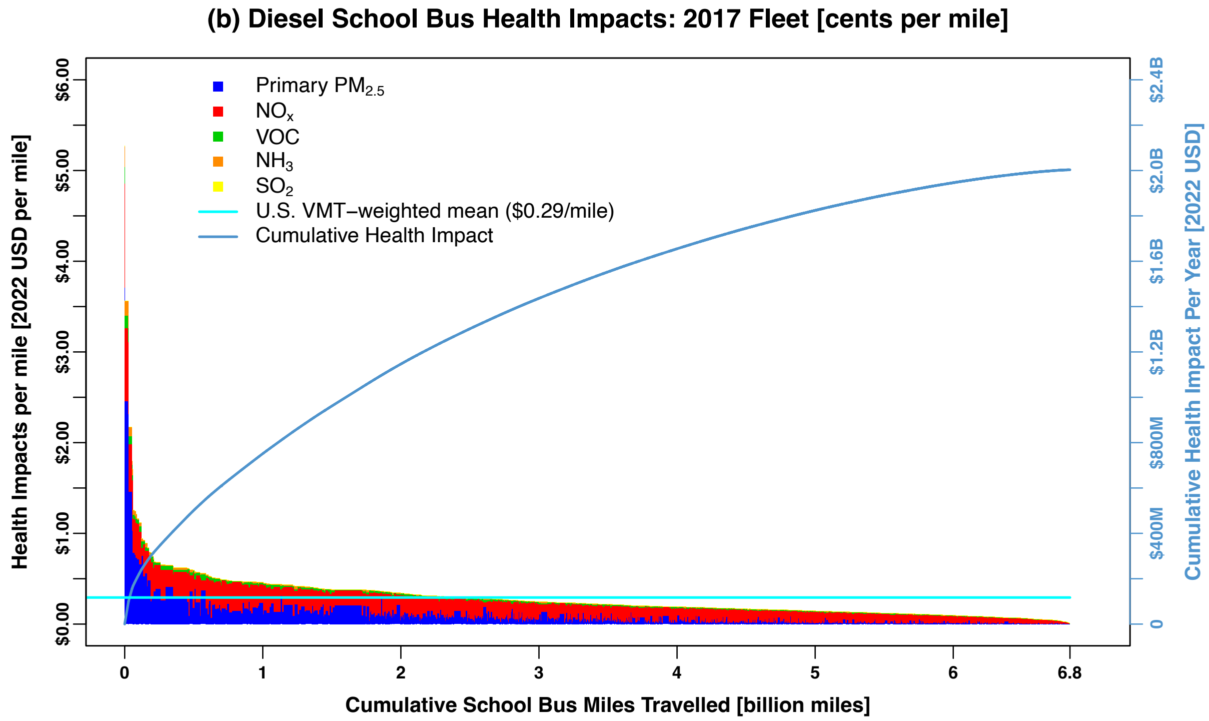

Figure S9. (a) Health benefits per of replacing diesel school buses (DSBs) in the fleet in 2017 with electric school buses (ESBs), by county. (b) Health impacts of DSBs in the fleet in 2017, by county. Counties are ordered from highest impact and benefit per mile (left) to lowest impact and benefit (right).

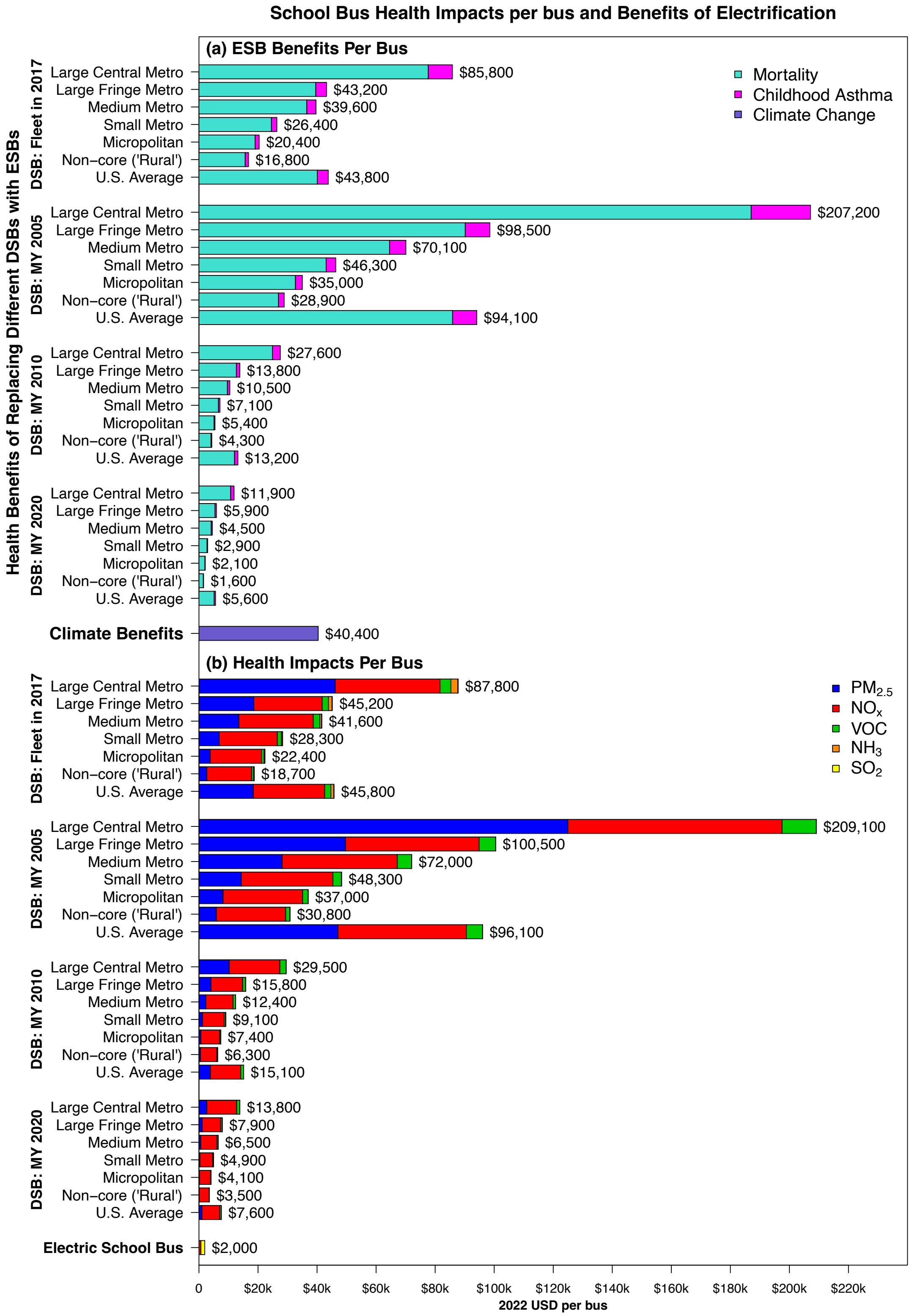

Figure S10. Per-vehicle health impacts and benefits of school bus electrification, by bus model year, driving location, pollutant species, and outcome. Locations are classified using NCHS's Urban-Rural classifications (25). DSB: Diesel School Bus. ESB: Electric School Bus.

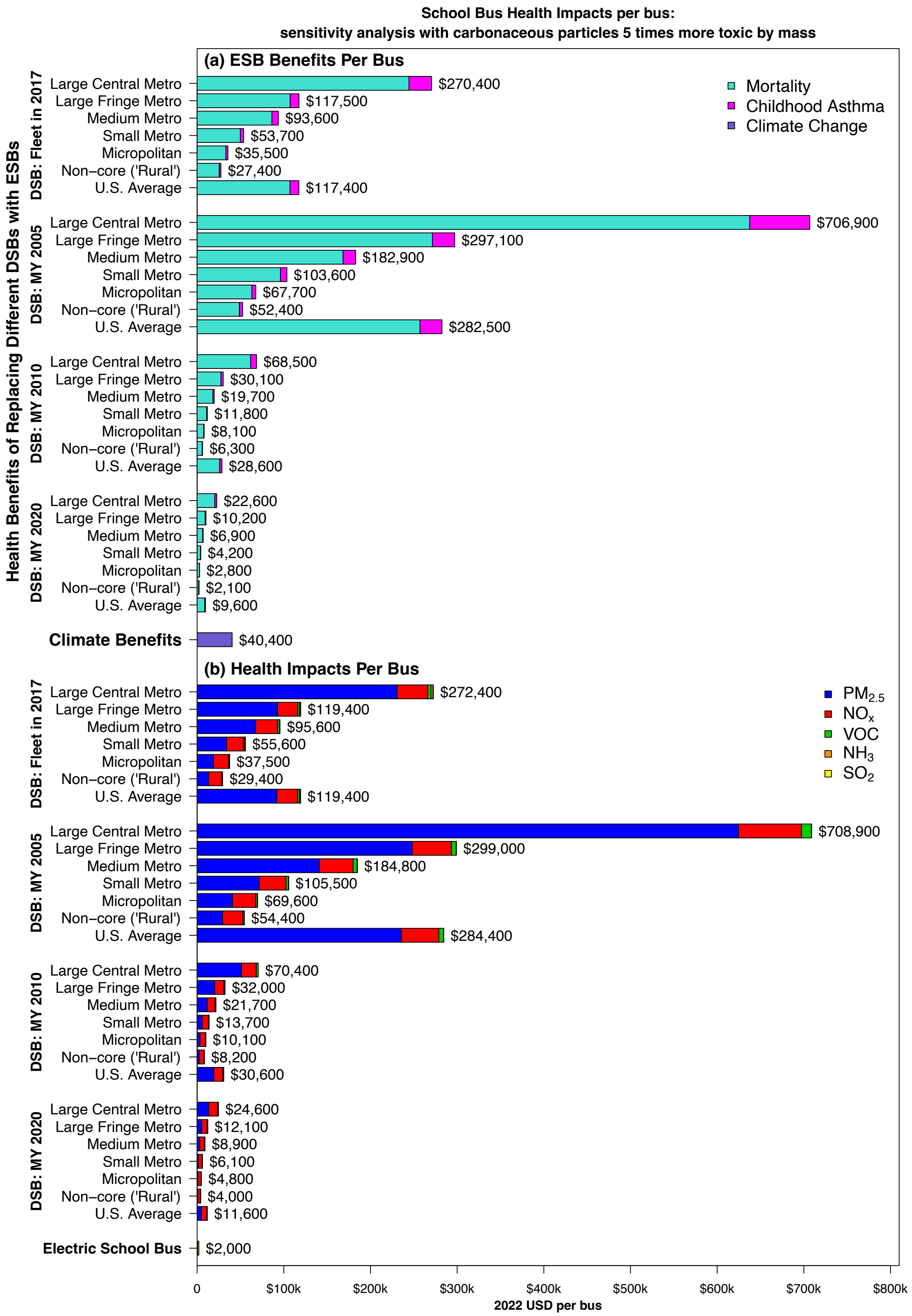

Figure S11. Per-vehicle health impacts and benefits of school bus electrification, by bus model year, driving location, pollutant species, and outcome: sensitivity analysis assuming carbonaceous particles are five times more toxic than the ambient mix, by mass. Locations are classified using NCHS's Urban-Rural classifications (25). DSB: Diesel School Bus. ESB: Electric School Bus.

3. Supplementary dataset descriptions for Datasets S1-S6 (separate files)

Dataset S1 (separate file). Supplementary results per mile, by county: Health benefits of electric school buses and health impacts of diesel school buses.

This dataset contains 3,116 rows x 73 columns.

Rows: rows 1-3,108 represent each of 3,108 counties in the contiguous United States. Rows 3,109-3,115 represent counties aggregated by National Center for Health Statistics' Urban-Rural classification (25). Row 3,116 represents the U.S. average. Aggregated results in rows 3,109-3,116 are weighted by vehicle miles travelled.

Columns:
Columns 1-7:
1) Row_Number: Row number.
2) Geography: Geography description.
3) FIPS_STCOU: FIPS state & county code, for counties (26).
4) County_Name: Name of the county (26).
5) FIPS_State: FIPS state code.
6) State_Name: State name.
7) NCHS_Urban_Rural_Classifications: Urban-Rural classification of the county from the National Center for Health Statistics (25).

Columns 8-73:
Columns 8-73 are named:
[Diesel Bus Model Year]_[Impact Type]_[Travel Unit]_[Pollutant]_[Health Outcome]
Where:
[Diesel Bus Model Year] is one of sets of emission factors considered according to diesel bus model year, i.e. 'NEI' (Fleet average in 2017 from EPA's National Emissions Inventory (2)), 'MY2005', 'MY2010', or 'MY2020';
[Impact Type] is either 'BEN' for benefits of electric school buses or 'DSB' for health impacts of diesel school buses;
[Travel Unit] is the travel unit, which is always 'Mile' (per mile) in Dataset S1;
[Pollutant] is one of five species, i.e., 'PM25' (Primary $PM_{2.5}$); 'SO2'; 'NOX'; 'NH3'; or 'VOC', or 'SUM' for the sum of all five species; and
[Health Outcome] is either 'Asthma', 'Deaths', or 'AllOutcomes' (for the sum of childhood asthma and mortality impacts).

Dataset S2 (separate file). Supplementary results per bus, by county: Health benefits of electric school buses and health impacts of diesel school buses.

This dataset contains 3,116 rows x 73 columns.

<u>Rows:</u> rows 1-3,108 represent each of 3,108 counties in the contiguous United States. Rows 3,109-3,115 represent counties aggregated by National Center for Health Statistics' Urban-Rural classification (25). Row 3,116 represents the U.S. average. Aggregated results in rows 3,109-3,116 are weighted by vehicle miles travelled.

<u>Columns:</u>
Columns 1-7:
1) Row_Number: Row number.
2) Geography: Geography description.
3) FIPS_STCOU: FIPS state & county code, for counties (26).
4) County_Name: Name of the county (26).
5) FIPS_State: FIPS state code.
6) State_Name: State name.
7) NCHS_Urban_Rural_Classifications: Urban-Rural classification of the county from the National Center for Health Statistics (25).

Columns 8-73:
Columns 8-73 are named:
[Diesel Bus Model Year]_[Impact Type]_[Travel Unit]_[Pollutant]_[Health Outcome]
Where:
[Diesel Bus Model Year] is one of sets of emission factors considered according to diesel bus model year, i.e. 'NEI' (Fleet average in 2017 from EPA's National Emissions Inventory (2)), 'MY2005', 'MY2010', or 'MY2020';
[Impact Type] is either 'BEN' for benefits of electric school buses or 'DSB' for health impacts of diesel school buses;
[Travel Unit] is the travel unit, which is always 'Bus (per bus) in Dataset S2;
[Pollutant] is one of five species, i.e., 'PM25' (Primary $PM_{2.5}$), 'SO2', 'NOX', 'NH3', or 'VOC', or 'SUM' for the sum of all five species; and
[Health Outcome] is either 'Asthma', 'Deaths', or 'AllOutcomes' (for the sum of childhood asthma and mortality impacts).

Dataset S3 (separate file). Supplementary results per mile, by school district: Health benefits of electric school buses and health impacts of diesel school buses.

This dataset contains 13,309 rows x 71 columns.

Rows: each row represents one school district

Columns:
Columns 1-5:
1) Row_Number: Row number.
2) LEAID: ID of local education agency (school district), according to the National Center for Education Statistics (20, 21).
3) NAME_LEA23: Name of local education agency (school district), according to the National Center for Education Statistics (20, 21).
4) FIPS_State: FIPS state code.
5) State_Name: State name.

Columns 6-71:
Columns 6-71 are named:
[Diesel Bus Model Year]_[Impact Type]_[Travel Unit]_[Pollutant]_[Health Outcome]
Where:
[Diesel Bus Model Year] is one of sets of emission factors considered according to diesel bus model year, i.e. 'NEI' (Fleet average in 2017 from EPA's National Emissions Inventory (2)), 'MY2005', 'MY2010', or 'MY2020';
[Impact Type] is either 'BEN' for benefits of electric school buses or 'DSB' for health impacts of diesel school buses;
[Travel Unit] is the travel unit, which is always 'Mile' (per mile) in Dataset S3;
[Pollutant] is one of five species, i.e., 'PM25' (Primary $PM_{2.5}$), 'SO2', 'NOX', 'NH3', or 'VOC', or 'SUM' for the sum of all five species; and
[Health Outcome] is either 'Asthma', 'Deaths', or 'AllOutcomes' (for the sum of childhood asthma and mortality impacts).

Dataset S4 (separate file). Supplementary results per bus, by school district: Health benefits of electric school buses and health impacts of diesel school buses.

This dataset contains 13,309 rows x 71 columns.

Rows: each row represents one school district

Columns:
Columns 1-5:
1) Row_Number: Row number.
2) LEAID: ID of local education agency (school district), according to the National Center for Education Statistics (20, 21).
3) NAME_LEA23: Name of local education agency (school district), according to the National Center for Education Statistics (20, 21).
4) FIPS_State: FIPS state code.
5) State_Name: State name.

Columns 6-71:
Columns 6-71 are named:
[Diesel Bus Model Year]_[Impact Type]_[Travel Unit]_[Pollutant]_[Health Outcome]
Where:
[Diesel Bus Model Year] is one of sets of emission factors considered according to diesel bus model year, i.e. 'NEI' (Fleet average in 2017 from EPA's National Emissions Inventory (2)), 'MY2005', 'MY2010', or 'MY2020';
[Impact Type] is either 'BEN' for benefits of electric school buses or 'DSB' for health impacts of diesel school buses;
[Travel Unit] is the travel unit, which is always 'Bus' (per bus) in Dataset S4;
[Pollutant] is one of five species, i.e., 'PM25' (Primary $PM_{2.5}$), 'SO2', 'NOX', 'NH3', or 'VOC', or 'SUM' for the sum of all five species; and
[Health Outcome] is either 'Asthma', 'Deaths', or 'AllOutcomes' (for the sum of childhood asthma and mortality impacts).

Dataset S5 (separate file). Supplementary results: marginal damages per metric ton of emissions at the ground-level, by county, pollutant and health outcome.

This dataset contains 3,108 rows x 22 columns.

Rows: Each row represents one county in the contiguous United States

Columns:
Columns 1-7:
1) Row_Number: Row number.
2) Geography: Geography description.
3) FIPS_STCOU: FIPS state & county code, for counties (26).
4) County_Name: Name of the county (26).
5) FIPS_State: FIPS state code.
6) State_Name: State name.
7) NCHS_Urban_Rural_Classifications: Urban-Rural classification of the county from the National Center for Health Statistics (25).

Columns 8-22:
Columns 8-73 are named:
MargDamages_[Pollutant]_[Health Outcome]
Where:
[Pollutant] is one of five species, i.e., 'PM25' (Primary $PM_{2.5}$); 'SO2', 'NOX', 'NH3', or 'VOC'; and [Health Outcome] is either 'Asthma', 'Deaths', or 'AllOutcomes' (for the sum of childhood asthma and mortality impacts).

Dataset S6 (separate file). Supplementary results: health impacts of electric school bus

This dataset contains 1 row x 19 columns.

Columns:
Columns 1:
1) Geography: Geography description. We only calculate the U.S. average impact for electric school buses.

Columns 2-19:
Columns 8-73 are named:
ESB_[Travel Unit]_[Pollutant]_[Health Outcome]
Where:
[Travel Unit] is either 'Mile' (for per-mile impacts) or 'BUS' (for per-bus impacts);
[Pollutant] is either 'SO2', 'NOX', or 'SUM' (for the sum of NO_x and SO_2 impacts); and
[Health Outcome] is either 'Asthma', 'Deaths', or 'AllOutcomes' (for the sum of childhood asthma and mortality impacts).

SI References

1. E. F. Choma *et al.*, Health benefits of decreases in on-road transportation emissions in the United States from 2008 to 2017. *Proceedings of the National Academy of Sciences* **118**, e2107402118 (2021).
2. U.S. Environmental Protection Agency, Data from the 2017 National Emissions Inventory (NEI). https://www.epa.gov/air-emissions-inventories/2017-national-emissions-inventory-nei-data. (Accessed 30 April 2021).
3. A. Burnham, MOVES3 Vehicle Operation Emission Factors. Argonne National Laboratory: Energy Systems Division, System Assessments Center. (2021). https://greet.es.anl.gov/files/update_moves3 (Accessed 7 August 2023).
4. U.S. Environmental Protection Agency, Data from Emissions & Generation Resource Integrated Database (eGRID): eGRID 2018. https://www.epa.gov/sites/production/files/2020-03/egrid2018_data_v2.xlsx (Accessed 22 April 2020).
5. E. F. Choma, J. S. Evans, J. K. Hammitt, J. A. Gómez-Ibáñez, J. D. Spengler, Assessing the health impacts of electric vehicles through air pollution in the United States. *Environment International* **144**, 106015 (2020).
6. U.S. Energy Information Administration, Annual Energy Outlook 2023: Table: Table 8. Electricity Supply, Disposition, Prices, and Emissions: Case: Reference Case. U.S. Energy Information Administration. https://www.eia.gov/outlooks/aeo/data/browser/#/?id=8-AEO2023&cases=ref2023&sourcekey=0 (Accessed 15 June 2023).
7. M. Levinson, P. Burgoyne-Allen, A. Huntington, N. Hutchinson (2023) Recommended total cost of ownership parameters for electric school buses: Summary of methods and data. Technical Note. Washington, DC: World Resources Institute. https://doi.org/10.46830/writn.22.00024 (Accessed 15 September 2023).
8. L. Lazer, L. Freehafer, J. Wang, https://datasets.wri.org/dataset/school_bus_fleets (Accessed 15 September 2023).
9. U.S. Department of Energy (n.d.) Where the Energy Goes: Electric Cars. https://www.fueleconomy.gov/feg/atv-ev.shtml (Accessed 24 August 2023)
10. U.S. Environmental Protection Agency (2014) Brake and Tire Wear Emissions from On-Road Vehicles in MOVES2014. EPA Document No. EPA-420-R-14-013. U.S. Environmental Protection Agency: Office of Transportation Air Quality: Assessments and Standards Division. https://nepis.epa.gov/Exe/ZyPDF.cgi/P100LCNE.PDF?Dockey=P100LCNE.PDF (Accessed 26 November 2023).
11. U.S. Environmental Protection Agency (2020) Brake and Tire Wear Emissions from Onroad Vehicles in MOVES3. U.S. Environmental Protection Agency: Assessment and Standards Division: Office of Transportation and Air Quality. EPA Document Number EPA-420-R-20-014. https://nepis.epa.gov/Exe/ZyPDF.cgi?Dockey=P1010M43.pdf (Accessed 26 November 2023).
12. R. A. Winer, X. Qin, T. Harrington, J. Moorman, H. Zahran, Asthma Incidence among Children and Adults: Findings from the Behavioral Risk Factor Surveillance System Asthma Call-back Survey—United States, 2006–2008. *Journal of Asthma* **49**, 16-22 (2012).
13. U.S. Centers for Disease Control and Prevention, Vintage 2020 Bridged-Race Postcensal Population Estimates: July 1, 2019. National Center for Health Statistics: National Vital Statistics System. https://www.cdc.gov/nchs/nvss/bridged_race/pcen_v2020_y19_txt.zip (Accessed 30 August 2023).
14. U.S. Centers for Disease Control and Prevention (2021) 2019 National Health Interview Survey (NHIS) Data: Table 4-1 Current Asthma Prevalence Percents by Age, United States: National Health Interview Survey, 2019. https://www.cdc.gov/asthma/nhis/2019/table4-1.htm (Accessed 5 August 2023).
15. A. L. Goodkind, C. W. Tessum, J. S. Coggins, J. D. Hill, J. D. Marshall, Fine-scale damage estimates of particulate matter air pollution reveal opportunities for location-

specific mitigation of emissions. *Proceedings of the National Academy of Sciences* **116**, 8775-8780 (2019).
16. A. L. Goodkind, C. W. Tessum, J. S. Coggins, J. D. Hill, J. D. Marshall, InMAP Source-Receptor Matrix (ISRM) dataset. https://doi.org/10.5281/zenodo.3590127 (Accessed 10 March 2021).
17. G. Appéré, D. Dussaux, A. Krupnick, M. Travers (2023) Valuing a reduction in the risk and severity of asthma. https://doi.org/10.1787/f289d29e-en (Accessed 20 June 2023).
18. E. Choma, Replication Data for: Choma, E. F., Evans, J. S., Gómez-Ibáñez, J. A., Di, Q., Schwartz, J., Hammitt, J. K., Spengler, J. D. (2021). "Health benefits of decreases in on-road transportation emissions in the United States from 2008 to 2017". Accepted for publication at Proceedings of the National Academy of Sciences of the United States of America. Harvard Dataverse. https://doi.org/10.7910/DVN/V3SXIM (Accessed 30 November 2021).
19. R. Burnett *et al.*, Global estimates of mortality associated with long-term exposure to outdoor fine particulate matter. *Proceedings of the National Academy of Sciences* **115**, 9592-9597 (2018).
20. National Center for Education Statistics, 2023 School District Geographic Relationship Files. https://nces.ed.gov/programs/edge/data/GRF23.zip (Accessed 19 February 2024).
21. D. Geverdt (2019) Education Demographic and Geographic Estimates Program (EDGE): School District Geographic Relationship Files User's Manual (NCES 2018-076). U.S. Department of Education. Washington, DC: National Center for Education Statistics. https://nces.ed.gov/programs/edge/Docs/EDGE_SDGRF_FILEDOC.pdf (Accessed 28 February 2024)
22. U.S. Census Bureau, 2018-2022 American Community Survey (ACS) 5-Year Estimates Detailed Tables. Table ID: ACSDT5Y2022.B01001. Table Title: Sex by Age. https://data.census.gov/table/ACSDT5Y2022.B01001?t=Age%20and%20Sex&g=010XX00US$1500000 (Accessed 19 February 2024).
23. U.S. Census Bureau, 2022 County Subdivision to 2020 Block Group for Connecticut. https://www2.census.gov/geo/docs/maps-data/data/rel2022/acs22_cousub22_blkgrp20_st09.txt (Accessed 20 February 2024).
24. U.S. Census Bureau, 2020 Census Demographic and Housing Characteristics File (DHC). Table ID: DECENNIALDHC2020.P1. Table Title: Title: TOTAL POPULATION. https://data.census.gov/table/DECENNIALDHC2020.P1?q=population&g=010XX00US$1500000&tid=DECENNIALDHCAS2020.P1 (Accessed 20 February 2024).
25. U.S. Centers for Disease Control and Prevention, NCHS Urban-Rural Classification Scheme for Counties. Centers for Disease Control and Prevention: National Center for Health Statistics. https://www.cdc.gov/nchs/data_access/urban_rural.htm (Accessed 15 June 2023).
26. U.S. Census Bureau, Population, Population Change, and Estimated Components of Population Change: April 1, 2010 to July 1, 2019 (CO-EST2019-alldata). https://www2.census.gov/programs-surveys/popest/datasets/2010-2019/counties/totals/co-est2019-alldata.csv (Accessed 3 May 2021).
